# Establishment of Reference Values for Selected Spatiotemporal and Kinematic Running Parameters in Healthy Adult Recreational Runners from Barranquilla, Colombia

**DOI:** 10.3390/jfmk11030350

**Published:** 2026-09-03

**Authors:** Enso Jiménez, Leila Marin, Raul Polo-Gallardo, Daniela Márquez, Otto Sierra, Javier Sanjuan

**Affiliations:** 1 Movement Analysis Laboratory, Biomedical Engineering Program, Universidad Simón Bolívar, Barranquilla 08001, Colombia; 2 Grupo de Investigación Muévete Caribe, Physiotherapy Program, Universidad Simón Bolívar, Barranquilla 08001, Colombia; leila.marin@unisimon.edu.co; 3 Nephrology/AudaciA Center for Artificial Intelligence and Robotics, Physical Therapy Program, Faculty of Health Sciences, Universidad Simón Bolívar, Barranquilla 08001, Colombia; raul.pologa@unisimon.edu.co; 4 Information Technologies Department, Institución Universitaria de Barranquilla, Barranquilla 080002, Colombia; daniela.marquez-s@uniminuto.edu.co; 5 GIMYP Research Unit, Department of Mechanical Engineering, Division of Engineering, Universidad del Norte, Km 5 Vía Puerto Colombia, Barranquilla 081007, Colombia; ottom@uninorte.edu.co

**Keywords:** running biomechanics, motion capture, running kinematics, reference values, gait analysis, sports medicine, injury prevention, spatiotemporal parameters, Barranquilla population

## Abstract

**Background and Objectives**: Running-related musculoskeletal injuries remain highly prevalent among recreational runners, and the absence of population-specific biomechanical reference values limits clinical and sports interpretation in Latin American populations. This study aimed to establish reference values for spatiotemporal and selected running kinematic/mechanical values (initial contact angle, vertical leg stiffness, and vertical oscillation) in healthy adult recreational runners from Barranquilla (Colombia) using three-dimensional motion capture. **Methods**: A cross-sectional observational study was conducted in 25 healthy adults (13 men, 12 women; mean age 32.7 ± 7.4 years, range 20–47 years). Running biomechanics were assessed using a BTS SMART-DX three-dimensional motion capture system and a Modified Helen Hayes marker model with 30 reflective markers. Participants completed treadmill running trials at 7, 9, 11, and 13 km/h. Key spatiotemporal parameters and global kinematic/mechanical metrics were processed using BTS Smart Clinic software. Reference values were defined using the 10th and 90th percentiles, stratified by sex and running speed, and accompanied by bootstrap 95% confidence intervals. Effects of sex and speed were tested by two-way ANOVA with partial eta squared and Holm-adjusted *p* values, with a linear mixed-effects model as a robustness check. **Results**: Motorized treadmill-specific biomechanical reference values (10th and 90th percentiles) were established and stratified strictly by running speed (7, 9, 11, and 13 km/h) and sex. Under the primary linear mixed-effects model, running speed significantly affected all ten evaluated biomechanical variables (all adjusted *p* < 0.001), including initial contact angle and vertical oscillation, confirming that speed-disaggregated estimates are mandatory for clinical interpretation. Conversely, sex effects were robust to multiplicity adjustment only for flight time and vertical leg stiffness. Compared with a European reference cohort, and after adjustment for sex, age, stature, body mass and speed through the published reference equations, this cohort showed a 6.6% shorter flight time, a 19.0% lower vertical oscillation, a 3.8% shorter stride length, and a 3.1% higher cadence. **Conclusions**: This study provides the preliminary regional reference database for running spatiotemporal and selected kinematic parameters in a cohort of healthy adult recreational runners from Barranquilla (Colombia). These preliminary values provide a baseline restricted to motorized treadmill running at fixed speeds; while they support standardized treadmill screening and rehabilitation tracking, their direct applicability to overground or self-paced running remains limited and warrants caution.

## 1. Introduction

Running is a fundamental form of human locomotion that requires the coordinated interaction of the musculoskeletal and neuromotor systems. As a complex motor task involving the synchronized movement of multiple joints and muscle groups, running has been extensively studied within the fields of biomechanics, sports science, rehabilitation, and clinical medicine due to its relevance for both health and athletic performance [1]. Running kinematics, defined as the quantitative description of body segment motion without considering the forces responsible for movement, has become an essential tool for identifying normal and pathological movement patterns and for understanding the biomechanical mechanisms underlying running performance and injury [2].

Running is one of the most popular physical activities worldwide and provides substantial health benefits, including reductions in all-cause mortality, cardiovascular disease risk, and disability [3]. Despite these benefits, running-related musculoskeletal injuries remain highly prevalent, particularly among recreational runners. Previous studies have reported annual injury rates approaching 70%, with the lower extremities representing the most frequently affected anatomical region [4]. Among these injuries, patellofemoral pain syndrome (PFPS) is one of the most common diagnoses, accounting for approximately 17% of all running-related injuries [5]. Several biomechanical and neuromuscular factors have been proposed to explain the development of PFPS, including altered lower-limb kinematics, abnormal mechanical loading, muscle weakness, and changes in muscle activation patterns during running [5].

Over the past decade, increasing evidence has suggested that proximal biomechanical factors play a critical role in the development of lower-extremity injuries. Clinical and biomechanical investigations have demonstrated that deficits in the control of the hip, pelvis, and trunk may alter tibiofemoral and patellofemoral joint mechanics across multiple planes of motion [6]. In particular, abnormal hip movement patterns have been associated with conditions such as anterior cruciate ligament injuries, iliotibial band syndrome, and patellofemoral pain [6]. Furthermore, sex-related differences have been reported, suggesting that female runners may be more susceptible to the influence of proximal biomechanical deficits [6]. Consequently, interventions focused on improving trunk stability, pelvic control, and hip function have been proposed as effective strategies for injury prevention and rehabilitation [6].

The clinical importance of running biomechanics extends beyond injury mechanisms. Reference kinematic values provide objective criteria for evaluating motor performance and identifying deviations associated with common running-related disorders, including iliotibial band syndrome, Achilles tendinopathy, patellofemoral pain syndrome, and plantar fasciitis. The availability of population-specific reference intervals allows clinicians, physiotherapists, sports physicians, and coaches to make more accurate decisions regarding injury prevention, rehabilitation, and performance optimization. Moreover, previous studies have demonstrated that running biomechanics vary according to age, with older runners exhibiting reduced preferred running speed, shorter stride lengths, longer stance times, and lower propulsive ground reaction forces compared with younger individuals [3]. Therefore, establishing reference values across adult age groups is essential for accurate biomechanical interpretation.

The quantitative assessment of running mechanics is currently performed using advanced motion analysis technologies. Among these, three-dimensional (3D) motion capture systems are considered the gold standard because of their high precision, reproducibility, and ability to characterize complex movement patterns [2]. These systems allow detailed assessment of temporal-spatial, kinematic, and kinetic parameters during walking and running and have become indispensable tools in both research and clinical biomechanics [2]. Riglet et al. recently developed a comprehensive 3D motion analysis dataset including healthy adults walking and running at different speeds on both treadmill and overground conditions, demonstrating the value of such databases for clinical research, biomechanical simulations, and artificial intelligence applications [2].

From a physiological and mechanical perspective, running involves the interaction of multiple biomechanical variables that influence movement efficiency and injury risk. Galdino et al. proposed hybrid nonlinear models to describe human locomotion by integrating concepts such as center-of-mass dynamics, energy efficiency, and ground reaction forces [7]. These models demonstrate that relatively small biomechanical alterations may substantially influence performance and injury susceptibility, emphasizing the importance of accurate and context-specific biomechanical measurements [7].

The high prevalence of running-related injuries has motivated extensive investigation into risk factors and preventive strategies. A systematic review by Van Gent et al. reported injury incidence rates ranging from 19% to 79%, identifying previous injury history, training volume, and running technique as major determinants of injury risk [4]. Similarly, Lee et al. demonstrated that biomechanical interventions such as customized foot orthoses may modify Achilles tendon loading and improve force distribution in runners with flat feet, potentially reducing the risk of overuse injuries [8]. These findings highlight the practical importance of understanding normal and abnormal running mechanics in different populations.

Despite the growing body of biomechanical research, the availability of reference datasets remains limited. Historically, many motion analysis laboratories have operated independently, restricting data sharing and limiting the development of robust biomechanical models [9]. To address this challenge, Ferber et al. recently published an open-access biomechanical dataset including 1798 healthy and injured individuals across different age groups and running conditions [9]. Such databases provide valuable resources for validating biomechanical methodologies, developing machine-learning algorithms, and identifying clinically relevant movement subgroups [9].

In parallel with advances in optical motion capture systems, portable technologies such as inertial measurement units (IMUs) have emerged as promising alternatives for biomechanical assessment. Asgari and Heller validated the use of sacral-mounted IMUs for reconstructing running kinematics in recreational runners and demonstrated their ability to identify distinct movement patterns using clustering techniques [10]. Nevertheless, despite the increasing popularity of wearable sensors, three-dimensional optical motion capture remains the gold standard for biomechanical analysis because of its superior accuracy and reliability [2].

Although international research has substantially advanced the understanding of running biomechanics, important knowledge gaps remain in Latin American populations. Most reference values currently used in clinical and sports settings originate from North American, European, or Australian cohorts [2,9]. Consequently, these values may not accurately represent populations with different anthropometric characteristics, environmental conditions, cultural behaviors, footwear habits, and training practices. This limitation is particularly relevant in Colombia and the Caribbean region, where biomechanical studies focused on running remain scarce.

Evidence from national studies suggests that contextual factors may significantly influence locomotor behavior. Guevara demonstrated that footwear characteristics can modify center-of-mass oscillation and energy expenditure during locomotion, indicating that biomechanical variables are not universally transferable across populations [11]. Likewise, research conducted in Colombia has reported associations between foot type and injury risk, emphasizing the importance of individualized biomechanical assessment in injury prevention strategies [12]. These findings reinforce the need for population-specific reference values capable of reflecting the unique characteristics of local runners. Furthermore, there is a relationship between the sociocultural environment and biomechanics, which finds solid scientific support in the international literature. In this regard, according to the research by Wallace et al. (2022) [13], the kinematics adopted by runners vary significantly across different societies depending on the cultural importance attributed to this activity. In groups where running is common and forms a central part of their collective identity, such as the Kalenjin of Kenya, a widespread adoption of efficient biomechanical techniques is observed, such as initial forefoot or midfoot contact and landing with the leg vertically aligned. In contrast, in populations where running is not culturally prioritized, such as the Tsimane of Bolivia or the Hadza of Tanzania, a suboptimal running pattern predominates almost unanimously, characterized by rearfoot strike impact.

Parallel to the influence of the sociocultural environment, the anthropometric characteristics of each population represent a decisive biological factor in the differentiation of locomotion patterns. From this perspective, the study by Patoz et al. (2022) [14], titled “Non-South East Asians have a better running economy and different anthropometrics and biomechanics than South East Asians,” reported that Southeast Asian runners were lighter, shorter, with lower BMI and shorter leg length than non-Southeast Asian runners. Likewise, the authors identified key biomechanical differences, such as a higher step frequency (cadence), shorter contact time, smaller foot contact angle, and less knee extension at takeoff in the Asian population. In summary, this article supports that anthropometric differences between populations are related to differences in running biomechanics and kinematics.

To justify and validate the sample size selected in this research, the systematic review and meta-analysis by Fukuchi et al. (2019) [15] on gait biomechanics in healthy adults was used as methodological support. In that study, specifically within Table 1 of that systematic review, it is evident that international research of high scientific quality on treadmills and level surfaces consistently uses small sample sizes for biomechanical and kinematic analyses. Among the studies compiled in that table, the motorized-treadmill protocols were based on samples of 20 young adult participants, one further treadmill study evaluated 15 participants, and the level-surface protocols supported their findings on samples of 14 participants.

Overall, the literature demonstrates a growing consensus regarding the importance of three-dimensional biomechanical analysis and the establishment of reference values for running mechanics. However, a substantial gap persists in studies involving Latin American populations, particularly individuals from Barranquilla. The absence of locally derived reference values limits the applicability of international databases and may compromise clinical interpretation and sports decision-making. Therefore, the objective of the present study was to establish reference spatio-temporal intervals and selected kinematic running values in healthy adult recreational runners from Barranquilla using three-dimensional motion capture technology. By generating a regional biomechanical reference database, this study aims to contribute to clinical assessment, injury prevention, rehabilitation, sports performance optimization, and future developments in biomechanical modeling and movement analysis.

## 2. Materials and Methods

### 2.1. Study Design

This study employed an observational, cross-sectional, descriptive design aimed at developing reference intervals for temporal, spatial, and selected kinematic/mechanical running variables in healthy adult recreational runners from Barranquilla. The study was conducted between 2025 and 2026 within the Biomechanics Laboratory of Universidad Simón Bolívar (Barranquilla, Colombia).

The primary outcome was the development of reference intervals for temporal, spatial, and kinematic running variables measured through three-dimensional motion capture technology. Secondary outcomes included the evaluation of the effects of running speed and sex on biomechanical parameters. The study was designed and reported according to current recommendations for observational biomechanical investigations and followed the ethical principles established in the Declaration of Helsinki.

### 2.2. Participants

A convenience sample of healthy recreational runners residing in Barranquilla was recruited through local running groups, universities, and community networks.

A convenience sample of healthy recreational runners residing in Barranquilla was recruited through an active community-targeted strategy. This included direct outreach to leaders of local recreational running clubs and public invitations shared on dedicated running social media groups. This approach aimed to recruit active runners who regularly train in urban spaces across the city. Initially, 32 volunteers were screened for participation. Seven individuals were excluded because of technical limitations associated with marker tracking during higher-speed trials, resulting in a final sample of 25 participants. The final cohort consisted of 52% men and 48% women, with ages ranging from 20 to 47 years.

Inclusion Criteria

Participants were eligible if they:

- Were between 18 and 65 years of age;
- Resided in Barranquilla;
- Had previous experience with recreational running, operationally defined as actively running for at least 12 consecutive months prior to the study, with a minimum frequency of two weekly training sessions, and no current or historical registration as an elite or professional athlete with any sports federation;
- Reported no musculoskeletal pain in the lower extremities during the previous three months;
- Were able to complete treadmill running trials safely;
- Signed an informed consent form.

Exclusion Criteria

Participants were excluded if they presented:

- Neurological disorders affecting gait or balance;
- Musculoskeletal injuries limiting running performance;
- Recent orthopedic surgery;
- Significant lower-limb length discrepancy;
- Cardiovascular contraindications identified through the Physical Activity Readiness Questionnaire (PAR-Q).
- Any condition capable of altering normal running mechanics.

### 2.3. Ethical Approval

The study protocol was reviewed and approved by the Research Ethics Committee of the Division of Health Sciences at Universidad del Norte (Act No. 341; 28 August 2025).

All participants received detailed information regarding the objectives, procedures, potential risks, and benefits of the investigation and provided written informed consent before participation. Confidentiality and anonymity were maintained throughout all stages of data collection, processing, and reporting.

### 2.4. Anthropometric Assessment

Prior to biomechanical testing, participants underwent anthropometric characterization. The following variables were recorded:

- Age (years);
- Height (cm);
- Body mass (kg);
- Body mass index (BMI; kg/m^2^);
- Percentage body fat (%);
- Skeletal muscle mass (kg).

Body composition measurements were obtained using an InBody 370S Body Composition Analyzer (InBody Co., Seoul, Republic of Korea). Additional anthropometric variables required for biomechanical modeling, including pelvic width, pelvic depth, lower-limb length, knee diameter, and intermalleolar distance, were automatically estimated by the BTS Smart Clinic software version 1.10 (BTS Bioengineering, Milan, Italy) following static calibration procedures.

### 2.5. Motion Capture System

Running kinematics were recorded using a BTS SMART-DX motion capture system (BTS Bioengineering, Milan, Italy). The system consisted of infrared optoelectronic cameras configured at an operational sampling frequency of 250 Hz (with a hardware acquisition frequency up to 340 Hz), spatial resolution of 0.1 mm, and the BTS Smart Clinic software platform. Before each acquisition session, the motion capture system was calibrated following the recommendation given in the acquisition procedure described in the BTS Davis Protocol, as specified in the manufacturer’s technical manual [16].

### 2.6. Marker Placement Protocol

**Figure 1:**
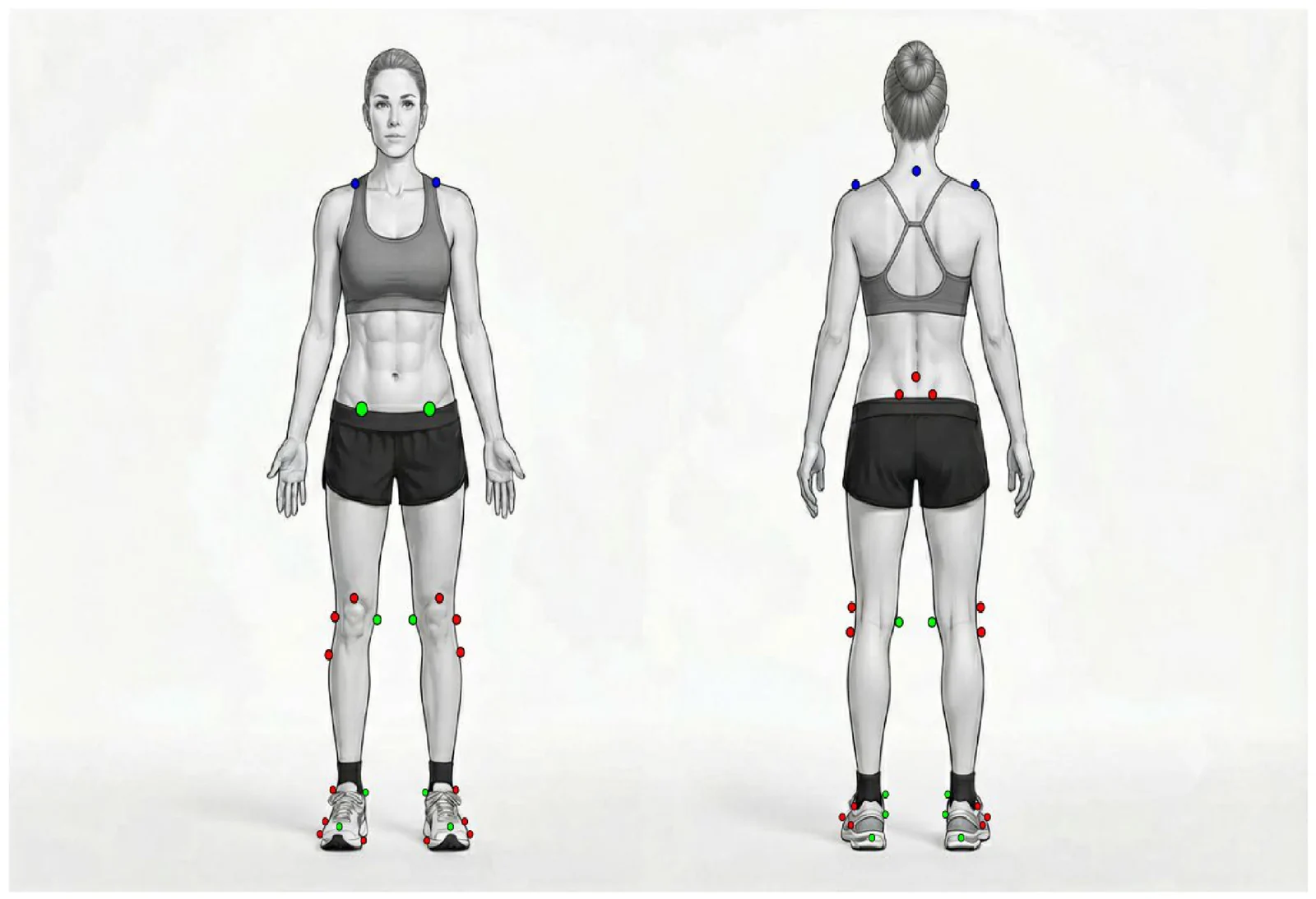
Marker placement in the Frontal and Posterior Plane, illustrating the complete, standardized 30-marker set used during the static calibration phase of the Modified Helen Hayes Model (MMHH). Green circles represent markers used only during the static calibration, and red/blue circles represent those retained for the dynamic running trials. Marker nomenclature and anatomical landmark definitions follow the Modified Helen Hayes implementation described in the BTS SMART-DX and SMART Clinic technical manual [16].

Thirty spherical reflective markers (15 mm diameter) were attached to specific anatomical landmarks using the Modified Helen Hayes Model (MMHH) during the static calibration phase. This protocol utilizes a 30-marker set for static calibration to accurately define participant-specific anthropometric parameters, after which 10 medial and pelvic markers are removed for the dynamic running trials to prevent marker contact and detachment, in strict accordance with the manufacturer’s guidelines and with the marker nomenclature, anatomical landmark definitions and segment-embedded coordinate systems specified in the BTS SMART-DX and SMART Clinic technical manual [16]. The kinematic model reconstructs eight rigid body segments (pelvis and trunk, plus thigh, shank and foot bilaterally) from the static calibration trial; joint centres are estimated from regression on the pelvic and knee/ankle landmark distances, and segment orientations are expressed following the Davis convention using an XYZ Cardan sequence. The complete marker set and its static/dynamic configuration are shown in Figure 1. This marker set has been extensively used in gait and running biomechanics and allows accurate reconstruction of lower-limb kinematics and temporal-spatial events. All marker placements were performed by the same trained investigator to minimize inter-rater variability.

### 2.7. Experimental Protocol

Participants attended a single testing session. Initially, a static calibration trial was recorded while participants stood comfortably in an anatomical position. The purpose of this trial was to define subject-specific anthropometric parameters and establish the biomechanical model. Subsequently, participants performed dynamic running trials on a motorized treadmill while wearing their habitual running shoes. Four running speeds were evaluated: 7 km/h, 9 km/h, 11 km/h and 13 km/h. These speeds were selected because they span the range of habitual training paces of recreational runners and bracket the mean preferred running speeds documented in the largest available reference cohort (10.5 ± 1.3 km/h in men and 9.0 ± 1.1 km/h in women) [17]; consequently, the resulting reference intervals cover the velocities at which recreational runners are most frequently assessed in clinical and sports settings, while the fixed-speed design permits direct between-participant comparison at matched absolute velocities.

Participants were allowed an acclimatization period of 1 min at each target speed before each recording to ensure stable running mechanics and familiarity with treadmill locomotion. Once running was stabilized, a continuous 3D motion capture acquisition was performed for 35 s. Running speed progression was implemented in ascending order. Trials were terminated if participants reported fatigue, discomfort, or inability to maintain the prescribed speed. Given the average cohort cadence of approximately 171 steps/min, this 35 s recording window yielded a mean of 49.8 ± 3.5 complete gait cycles per limb at each speed condition (range, 44–62 cycles; 47.6 ± 2.4 at 7 km/h and 52.3 ± 3.6 at 13 km/h), corresponding to approximately 100 steps per trial. All valid consecutive cycles captured within this window were processed and averaged for each subject to ensure exceptional biomechanical reliability. One participant was unable to complete the 13 km/h condition, and one participant experienced a software issue at one speed, resulting in 98 valid running reports across all speed categories.

### 2.8. Data Processing

Marker trajectories were processed using BTS Smart Clinic software. Raw 3D marker coordinates were preprocessed and smoothed within the BTS Smart Clinic software using a fourth-order, zero-lag, low-pass Butterworth filter with a cutoff frequency of 10 Hz. This cutoff was not arbitrary: for three-dimensional lower-extremity marker trajectories acquired at 250 Hz during running, both residual analysis and Fourier spectral analysis retaining 95% of the signal power converge on an optimal cutoff of approximately 10 Hz [18,19]. At this cutoff, displacement and first-derivative waveforms are free of high-frequency noise while the true signal remains minimally attenuated, whereas higher cutoffs (≥15 Hz) leave residual noise and lower cutoffs (≤5 Hz) progressively distort the kinematic waveform [19]. From the software, running events such as heel strike, toe-off, among others, were marked following the filtered trajectories. The biomechanical variables were extracted:

**Temporal Variables and Spatial Variables:** Running cycle time (s); Stance time (s); Flight time (s); Stance phase (% cycle); Flight phase (% cycle) and Stride length (m).

**Kinematic Variables:** Initial contact angle (°); Vertical oscillation (m) and Cadence (steps/min).

**Kinetic Variable:** Vertical leg stiffness (kN/m).

**Initial Contact Angle (°) (Foot Strike Angle):** This variable represents the sagittal plane angle and segmental angle of the foot segment relative to the horizontal laboratory floor (and not a joint angle such as hip, knee, or ankle flexion) at the exact frame of initial contact (heel strike). It was calculated using the vector defined by the calcaneus (CAL) and the second metatarsal head (MT2) markers relative to the treadmill surface:

$$\theta_{contact}=arctan\left(\frac{z_{MT2}-z_{CAL}}{x_{MT2}-x_{CAL}}\right)$$

where z represents the vertical coordinate and x the anteroposterior coordinate of the markers. A positive angle denotes a dorsiflexed foot position relative to the horizontal, which is indicative of a rearfoot strike pattern, whereas negative values indicate a plantarflexed foot position at contact, characteristic of midfoot or forefoot strike patterns.

Vertical Oscillation of the Center of Mass (m): Rather than pelvic displacement, this variable measures the vertical excursion of the body’s total Center of Mass (CoM). The 3D trajectory of the CoM (CoMz) was dynamically estimated at each frame by the BTS Smart Clinic software through a multi-segment model based on the 30-marker Modified Helen Hayes protocol. Vertical oscillation (VO) was defined as:

$$VO=z_{max}-z_{min}$$

where zmax and zmin represent the maximum and minimum vertical coordinates of the estimated CoM trajectory during a complete running cycle.

Vertical Leg Stiffness (*k_leg_*, kN/m): Since data collection was restricted to 3D kinematics without direct ground reaction force (GRF) measurements, leg stiffness was estimated using the mathematically validated sine-wave mass-spring model proposed by Morin et al. (2005) [20]. This model calculates the peak vertical ground reaction force (*F*_max_) and leg compression (Δ*y*) during the stance phase using body mass, treadmill speed, and temporal-spatial kinematics:

$$F_{max}\;=\;m\;\cdot \;g\;\cdot \;\frac{\pi }{2}\left(\frac{t_{f}}{t_{c}}\;+\;1\right)$$

$$\Delta y=\Delta z+L\left(1-cos\;\theta \right)$$

$$\theta =arcsin\left(\frac{v\;\cdot \;t_{c}}{2L}\right)$$

where

○ m is the participant’s body mass (kg).
○ g is the acceleration due to gravity (9.81 m/s^2^).
○ *t_f_* is flight time (s) and *t_c_* is contact/stance time (s).
○ Δ*z* is the vertical displacement of the CoM during the stance phase (m).
○ *L* is the initial leg length (m), measured from the greater trochanter to the floor during static calibration.
○ *v* is the treadmill running speed (m/s).

Finally, vertical leg stiffness was computed as:

$$k_{leg}\;=\;\frac{F_{max}}{\Delta y}$$

### 2.9. Statistical Analysis

Statistical analyses were performed using Python, utilizing libraries such as seaborn, pandas, and ols, among others. Continuous variables were summarized using means and standard deviations, whereas categorical variables were described using frequencies and percentages. Distributional assumptions were verified with the Shapiro–Wilk test applied both to the pooled distribution and within each sex × speed stratum; stride length, vertical oscillation and cadence departed from normality (*p* < 0.01) and were therefore additionally handled with distribution-free procedures, whereas the remaining seven variables were compatible with a parametric treatment (*p* > 0.05).

Reference intervals were established through percentile analysis. Following the consensus and methodologies reported in the scientific literature for the establishment of reference values of human locomotion, such as the study by Kim et al. (2025) on gait parameters, reference cutoff values are formally defined based on the 10th (P10) and 90th (P90) percentiles of each parameter under analysis [21]. This methodological approach allows delimiting a clinical reference interval that contains the central 80% of the healthy population, offering a much more robust, reliable, and representative clinical decision-making framework. In this sense, values that fall below the 10th percentile or above the 90th percentile for each kinematic or kinetic variable correspond to atypical or extreme biomechanical values of the distribution. Values outside this interval were interpreted as deviations from the central distribution of healthy recreational runners.

Because percentile estimates obtained from moderate sample sizes carry non-negligible sampling uncertainty, 95% confidence intervals for the P10, P50 and P90 estimates of every sex × speed stratum were computed by bias-corrected and accelerated (BCa) bootstrapping with 10,000 resamples [22]. These intervals are reported in Supplementary Table S2 and should be taken into account whenever an individual runner is classified as falling outside the reference limits.

The effects of sex and running speed on each biomechanical variable were examined with a two-way factorial ANOVA (sex × speed), including the interaction term. Residual normality and homogeneity of variances were assessed with the Shapiro–Wilk and Levene tests, respectively. Significant effects were followed by Tukey HSD post hoc tests for variables meeting normality and by Dunn tests for the remaining variables. Effect sizes were reported for every inferential test: partial eta squared (η^2^p) for each ANOVA term, with the conventional thresholds of 0.01, 0.06 and 0.14 for small, medium and large effects, and Cohen’s dz for the pairwise speed contrasts. Because 30 omnibus tests (10 variables × 3 model terms) and six pairwise speed contrasts per variable were performed, all *p* values were adjusted for multiplicity using the Holm–Bonferroni sequentially rejective procedure [23] and, as a less conservative alternative that controls the false discovery rate, the Benjamini–Hochberg procedure [24]. Both unadjusted and adjusted *p* values are reported so that readers may judge the robustness of each finding. Statistical significance was set at *p* < 0.05.

Two additional analyses were performed to verify the robustness of the inferential results. First, because each participant contributed up to four speed conditions, the factorial model was refitted as a linear mixed-effects model including a random intercept for participant, with fixed effects tested by likelihood-ratio tests and the intraclass correlation coefficient (ICC) computed to quantify within-participant dependence. Second, a sensitivity analysis was performed after excluding the two participants who did not complete the full four-speed series, in order to confirm that the descriptive estimates and reference percentiles were not driven by incomplete observation sets. All analyses were performed in Python 3.10 using pandas, NumPy, SciPy, statsmodels and seaborn.

## 3. Results

### 3.1. Participant Characteristics

Thirty-two recreational runners were initially recruited for participation. Seven individuals were excluded from the final analysis because reflective markers became partially detached during high-speed running trials, compromising the quality of motion-capture tracking. Consequently, 25 participants were included in the final dataset.

Considering that four running speeds were evaluated for each participant, a total of 98 valid running reports were analyzed. One participant was unable to complete the highest-speed condition (13 km/h), and one report was excluded because of software processing issues. The final sample demonstrated a balanced sex distribution, with 52% men and 48% women. Participants had a mean age of 32.7 ± 7.4 years (range, 20–47 years; men, 31.8 ± 7.5 years; women, 33.6 ± 7.5 years), a mean height of 167.8 ± 8.2 cm, a mean body mass of 69.2 ± 9.3 kg, a mean body mass index of 24.5 ± 2.3 kg/m^2^, a mean body fat of 23.7 ± 8.4% and a mean skeletal muscle mass of 29.8 ± 6.5 kg (see Table 1 for the full sex-stratified demographic and anthropometric description). By inclusion criteria, all participants had practised recreational running for at least 12 consecutive months with a minimum of two weekly training sessions, and none was federated as an elite or professional athlete; detailed training-load descriptors such as weekly mileage and years of running experience were not systematically recorded, which is acknowledged in Section 4.5. Across the 98 valid reports, a mean of 49.8 ± 3.5 complete gait cycles per limb (range, 44–62) was available for averaging at each speed condition, and the number of valid observations per sex × speed stratum ranged from 11 to 13.

**Table 1:** Participant characteristics and overall biomechanical variables.

Variable	Total (*n* = 25)	Men (*n* = 13)	Women (*n* = 12)
Age (years) ^1^	20–47	20–41	21–47
Age (years), mean ± SD	32.7 ± 7.4	31.8 ± 7.5	33.6 ± 7.5
Height (cm)	167.76 ± 8.21	174.46 ± 3.99	160.50 ± 4.36
Body mass (kg)	69.15 ± 9.31	76.39 ± 5.36	61.30 ± 5.36
Body mass index (kg/m^2^)	24.52 ± 2.27	25.15 ± 2.20	23.83 ± 2.23
Body fat (%)	23.68 ± 8.35	18.23 ± 6.11	29.58 ± 6.18
Skeletal muscle mass (kg)	29.84 ± 6.48	35.59 ± 2.16	23.60 ± 2.24
Running cycle time (s)	0.706 ± 0.047	0.723 ± 0.037	0.688 ± 0.050
Stance time (s)	0.290 ± 0.039	0.292 ± 0.036	0.289 ± 0.042
Flight time (s)	0.416 ± 0.031	0.431 ± 0.031	0.399 ± 0.022
Stance phase (% cycle)	41.04 ± 3.79	40.36 ± 3.97	41.78 ± 3.48
Flight phase (% cycle)	58.97 ± 3.80	59.64 ± 3.97	58.25 ± 3.49
Stride length (m)	1.94 ± 0.39	2.04 ± 0.41	1.88 ± 0.36
Initial contact angle (°)	11.18 ± 5.88	12.08 ± 4.83	10.20 ± 6.75
Vertical leg stiffness (kN/m)	13.61 ± 2.73	14.91 ± 2.47	12.20 ± 2.28
Vertical oscillation (m)	0.06 ± 0.01	0.06 ± 0.01	0.06 ± 0.02
Cadence (steps/min)	170.72 ± 12.06	166.42 ± 8.71	175.39 ± 13.46

Values are mean ± standard deviation unless otherwise indicated. ^1^ Age is reported as range (minimum–maximum). *n* = 25 (Men = 13, 52%; Women = 12, 48%). Pooled biomechanical variables correspond to all running speeds combined and are presented solely for sample characterization; due to the strong speed-dependency of running kinematics, these pooled averages must not be used as clinical or normative reference standards (practitioners must refer strictly to the speed-disaggregated percentiles in Table 2). BMI, body mass index.

**Table 2:** Percentiles (P10, P25, P50, P75, and P90) for biomechanical variables according to sex and running speed.

		Men						Women					
Variable	Speed (km/h)	*n*	P10	P25	P50	P75	P90	*n*	P10	P25	P50	P75	P90
Running cycle time (s)	7	13	0.72	0.73	0.75	0.76	0.77	12	0.68	0.70	0.72	0.77	0.78
	9	12	0.71	0.72	0.74	0.76	0.77	12	0.65	0.68	0.70	0.74	0.75
	11	13	0.69	0.70	0.72	0.73	0.75	12	0.62	0.66	0.68	0.70	0.71
	13	13	0.66	0.68	0.69	0.72	0.73	11	0.58	0.64	0.65	0.67	0.68
Stance time (s)	7	13	0.29	0.31	0.34	0.35	0.36	12	0.31	0.32	0.33	0.35	0.38
	9	12	0.28	0.29	0.30	0.32	0.33	12	0.28	0.28	0.30	0.32	0.33
	11	13	0.26	0.26	0.28	0.29	0.29	12	0.25	0.26	0.28	0.28	0.30
	13	13	0.24	0.25	0.26	0.26	0.28	11	0.20	0.24	0.25	0.26	0.27
Flight time (s)	7	13	0.37	0.38	0.40	0.42	0.44	12	0.37	0.38	0.39	0.40	0.41
	9	12	0.41	0.42	0.44	0.45	0.47	12	0.37	0.39	0.41	0.42	0.42
	11	13	0.42	0.43	0.44	0.44	0.47	12	0.38	0.39	0.41	0.42	0.42
	13	13	0.42	0.43	0.44	0.45	0.47	11	0.38	0.39	0.40	0.42	0.42
Stance phase (% cycle)	7	13	40.21	44.76	46.08	47.24	48.96	12	43.39	45.02	46.05	47.36	48.49
	9	12	37.60	40.38	41.80	42.30	42.61	12	40.99	41.20	42.21	44.14	44.52
	11	13	35.67	38.02	39.38	40.17	40.39	12	38.60	38.97	39.86	41.63	42.25
	13	13	34.98	36.40	37.34	38.14	38.42	11	34.90	37.03	37.80	40.02	40.50
Flight phase (% cycle)	7	13	51.04	52.76	53.92	55.23	59.81	12	51.51	52.63	53.95	54.99	56.61
	9	12	57.40	57.70	58.20	59.62	62.41	12	55.48	55.86	57.79	58.80	59.02
	11	13	59.62	59.82	60.62	61.99	64.33	12	57.76	58.38	60.14	61.03	61.40
	13	13	61.58	61.86	62.64	63.60	65.03	11	59.52	60.46	62.20	62.97	65.10
Stride length (m)	7	13	1.39	1.41	1.46	1.47	1.50	12	1.32	1.35	1.40	1.50	1.52
	9	12	1.78	1.81	1.85	1.90	1.93	12	1.62	1.69	1.74	1.85	1.88
	11	13	2.10	2.13	2.19	2.24	2.27	12	1.92	2.02	2.06	2.15	2.18
	13	13	2.39	2.44	2.50	2.61	2.63	11	2.08	2.31	2.36	2.43	2.46
Initial contact angle (°)	7	13	4.16	5.35	9.75	10.90	13.19	12	1.53	4.15	6.75	11.45	16.61
	9	12	5.94	6.62	12.08	14.62	17.58	12	1.55	3.91	8.32	12.41	19.50
	11	13	8.38	9.95	13.85	14.90	18.48	12	5.24	7.01	10.58	15.19	20.36
	13	13	10.11	12.55	14.85	17.40	19.99	11	4.30	7.88	11.05	15.12	19.35
Vertical leg stiffness (kN/m)	7	13	10.97	11.44	12.90	16.25	16.89	12	8.49	9.52	10.00	11.14	11.91
	9	12	12.14	12.54	13.26	14.86	16.78	12	10.07	10.22	11.10	13.20	13.90
	11	13	13.73	14.00	14.82	16.40	18.78	12	10.82	11.46	12.07	14.69	15.37
	13	13	14.12	15.11	15.71	18.19	19.66	11	11.60	12.88	14.14	15.58	16.43
Vertical oscillation (m)	7	13	0.03	0.06	0.06	0.07	0.08	12	0.05	0.06	0.07	0.07	0.08
	9	12	0.05	0.06	0.06	0.07	0.08	12	0.06	0.06	0.07	0.07	0.08
	11	13	0.05	0.05	0.06	0.07	0.08	12	0.05	0.06	0.06	0.07	0.07
	13	13	0.05	0.05	0.06	0.06	0.07	11	0.04	0.04	0.06	0.06	0.06
Cadence (steps/min)	7	13	155.52	159.00	159.60	165.00	167.76	12	153.66	155.55	166.50	171.90	176.22
	9	12	155.46	157.80	162.00	165.75	168.36	12	159.78	161.85	171.90	177.45	185.10
	11	13	161.40	163.80	167.40	171.60	174.84	12	168.12	171.00	177.90	181.20	191.10
	13	13	164.52	165.60	173.40	177.60	181.68	11	176.40	178.50	183.60	187.80	207.60

Note. P10, P25, P50, P75, and P90 denote the 10th, 25th, 50th, 75th, and 90th percentiles, respectively. P50 is the median; *n* indicates the number of valid observations within each sex and speed stratum. For bilateral variables, right- and left-side values were averaged before percentile estimation. Given the stratum sizes (*n* = 11–13), these percentile estimates carry appreciable sampling uncertainty; bias-corrected and accelerated bootstrap 95% confidence intervals for P10, P50 and P90 are provided in Supplementary Table S2 and should be consulted before classifying an individual runner as atypical.

As expected, men showed greater height, body mass, and skeletal muscle mass, and lower body fat than women, whereas BMI remained within the normal range in both sexes (around 24–25 kg/m^2^), as summarized in the overall profile presented in Table 1. Although pooled cohort averages across all evaluated speeds combined are presented in Table 1 to describe the general sample characteristics (e.g., overall running cycle time of 0.71 ± 0.05 s, stance time of 0.29 ± 0.04 s, and flight time of 0.42 ± 0.03 s), these pooled biomechanical variables possess restricted clinical and functional utility because of the strong speed-dependency of running kinematics. Therefore, these combined averages must not be interpreted as normative reference standards. Practitioners and researchers must refer strictly to the speed- and sex-disaggregated percentile values presented in Table 2, which provide the mathematically and clinically appropriate framework for biomechanical interpretation across distinct running paces.

These values provide the first descriptive biomechanical profile of healthy recreational runners from Barranquilla.

### 3.2. Reference Intervals

Reference values were established using percentile-based analyses. Following conventional approaches in clinical biomechanics, the interval between the 10th percentile (P10) and the 90th percentile (P90) was considered the reference interval for each variable, which are fully detailed and stratified by sex and running speed in Table 2.

Values below P10 or above P90 were interpreted as deviations from the central distribution of healthy recreational runners and may therefore warrant further biomechanical assessment in clinical or sports settings. Reference intervals were stratified according to sex and running speed, allowing more precise interpretation of biomechanical performance under different locomotor demands.

Temporal and spatial variables showed the clearest speed dependence: running cycle time, stance time and stance-phase percentage decreased as speed increased, whereas flight-phase percentage, stride length and cadence increased. Stride length showed the largest relative change, nearly doubling from 7 to 13 km/h in both sexes. Vertical leg stiffness rose with speed and was consistently higher in men, whereas women maintained a higher cadence at all speeds. Initial contact angle increased with speed but with wide interquartile ranges, and vertical oscillation remained essentially stable (around 0.05–0.07 m), consistent with the non-significant ANOVA effect for these two variables. Together, these intervals support the use of speed- and sex-specific reference ranges rather than a single pooled reference, allowing precise comparison across all evaluated percentiles (Table 2).

The percentile-based approach provides practical clinical utility because it allows practitioners to compare an individual runner against a locally derived reference population. Such information may facilitate injury-risk screening, rehabilitation monitoring, and performance assessment.

A sensitivity analysis excluding the two participants with an incomplete four-speed series (one who did not complete the 13 km/h condition and one whose report at 9 km/h was lost to a software processing error) (leaving 92 valid reports from 23 participants) confirmed the stability of the reference estimates: pooled means changed by less than 3% for every variable (largest change, −2.6% for the initial contact angle; all remaining variables ≤ 1.1%), and the corresponding P10 and P90 limits were essentially unchanged. The reference intervals reported in Table 2 are therefore not driven by the incomplete observation sets.

### 3.3. Distribution of Biomechanical Variables

The distribution of temporal, spatial, and kinematic variables according to sex and running speed is presented in Figure 2, Figure 3, Figure 4, Figure 5, Figure 6, Figure 7, Figure 8, Figure 9, Figure 10 and Figure 11.

The figures show a pattern consistent with the two-way ANOVA. Running speed significantly modified all ten variables: running cycle time and stance time decreased as speed increased, whereas stride length, flight-phase percentage and cadence increased progressively. Stride length and the stance- and flight-phase percentages differed across all speed pairs, while running cycle time, vertical leg stiffness and cadence differed mainly between the extreme speeds (7 vs. 13 km/h). By sex, men showed greater stride length and vertical leg stiffness, and women a higher cadence; stance time, initial contact angle and vertical oscillation did not differ by sex. The sex × speed interaction was not significant for any variable, so men and women responded similarly to increasing speed. The outliers visible in some boxplots represented less than 3.1% of observations and were retained as inter-individual variability.

### 3.4. Summary of ANOVA Findings

A two-way factorial ANOVA (sex × speed) including the interaction term was used as the primary inferential analysis. The effect of running SPEED was significant in all ten variables before adjustment (stance time *p* < 0.001; initial contact angle *p* = 0.046; vertical oscillation *p* = 0.040) and the associated effect sizes were large for the spatiotemporal descriptors: stride length η^2^p = 0.930, flight-phase percentage η^2^p = 0.644, stance time η^2^p = 0.644, stance-phase percentage η^2^p = 0.641, running cycle time η^2^p = 0.337, cadence η^2^p = 0.324 and vertical leg stiffness η^2^p = 0.296, against small effects for flight time (η^2^p = 0.110), vertical oscillation (η^2^p = 0.088) and initial contact angle (η^2^p = 0.085). After Holm–Bonferroni adjustment across the 30 omnibus tests, the speed effect remained significant for seven of the ten variables (all adjusted *p* < 0.001), whereas it no longer reached the conventional threshold for flight time (adjusted *p* = 0.232), vertical oscillation (adjusted *p* = 0.607) and initial contact angle (adjusted *p* = 0.639); under the less conservative Benjamini–Hochberg criterion the flight-time effect was retained (adjusted *p* = 0.029) while vertical oscillation (0.076) and initial contact angle (0.081) remained borderline. The effect of SEX was significant in seven variables before adjustment and non-significant in three (stance time, initial contact angle and vertical oscillation); after Holm adjustment it persisted for vertical leg stiffness (η^2^p = 0.313), flight time (η^2^p = 0.290), stride length (η^2^p = 0.217), running cycle time (η^2^p = 0.212) and cadence (η^2^p = 0.208), while the smaller effects on stance- and flight-phase percentages (η^2^p = 0.082 and 0.080) survived only the Benjamini–Hochberg criterion. The sex × speed interaction was not significant for any variable in the factorial model (all *p* > 0.05).

Because each participant contributed up to four speed conditions, the factorial model was refitted as a linear mixed-effects model with a random intercept per participant. The within-participant intraclass correlation was high for every variable (ICC = 0.71–0.88), confirming that a substantial share of the total variance is attributable to stable inter-individual differences. Under this framework the effect of speed was significant for all ten variables after Holm adjustment (all adjusted *p* < 0.001), including initial contact angle and vertical oscillation, indicating that the loss of significance for these two variables in the multiplicity-adjusted factorial model reflects the conservative treatment of between-participant variance rather than an absence of a speed effect. Conversely, the sex effect was attenuated once inter-individual dependence was modelled and remained significant after Holm adjustment only for flight time (adjusted *p* = 0.037) and vertical leg stiffness (adjusted *p* = 0.022), while sex × speed interactions emerged for running cycle time, flight time, stride length, vertical oscillation and cadence. These convergences and divergences are considered when interpreting the findings and are addressed in Section 4.5.

### 3.5. Comparison with International Reference Values

To place the present reference values in an international context, the three spatiotemporal parameters operationally comparable with the large European reference cohort of Malisoux et al. [17] (flight time, vertical oscillation of the centre of mass, and cadence) were contrasted with that dataset. To avoid pseudo-replication, each participant contributed a single value per parameter (the mean across the speed conditions completed), so that the comparison was performed at the participant level (*n* = 25) against the reference sample (*n* = 860) using Welch’s *t*-test, with Hedges’ g as the effect-size estimate (Table 3A).

Because the two cohorts differ in age, stature, body mass and testing speed, a second, fully adjusted comparison was performed. The reference regression equations published by Malisoux et al. [17] were applied to each observation of the present cohort using that participant’s own sex, age, body mass, height and treadmill speed, yielding an individually predicted value under the European reference model. Observed and predicted values were then compared with paired *t*-tests and Cohen’s dz (Table 3B). Stride length was included in this adjusted analysis after converting the reference step-length equation to a stride basis; vertical leg stiffness was not compared because the two studies use non-equivalent operational definitions.

In the unadjusted comparison Barranquilla cohort showed a shorter flight time (0.415 ± 0.029 s vs. 0.444 ± 0.038 s; mean difference −0.029 s, 95% CI −0.041 to −0.017; *p* < 0.001; g = −0.76), a lower vertical oscillation (0.062 ± 0.013 m vs. 0.077 ± 0.012 m; −0.015 m, 95% CI −0.020 to −0.009; *p* < 0.001; g = −1.22) and a higher cadence (170.9 ± 9.9 vs. 164.0 ± 9.0 steps/min; +6.89 steps/min, 95% CI +2.75 to +11.02; *p* = 0.002; g = +0.76). All three differences persisted after full adjustment through the reference equations: flight time was 6.6% shorter than predicted (dz = −1.44; *p* < 0.001), vertical oscillation 19.0% lower (dz = −1.11; *p* < 0.001), cadence 3.1% higher (dz = +0.67; *p* = 0.003) and stride length 3.8% shorter (dz = −0.92; *p* < 0.001).

## 4. Discussion

The primary objective of this study was to establish reference spatiotemporal and selected kinematic/mechanical running values for healthy adult recreational runners from Barranquilla using three-dimensional motion capture technology. This investigation represents the first study to generate a regional biomechanical reference database for running kinematics in this population. The results provide reference percentile-based intervals for temporal, spatial, and kinematic variables.

### 4.1. Establishment of Population-Specific Reference Values

The development of reference biomechanical values constitutes one of the most important contributions of this investigation. Running biomechanics is frequently evaluated using reference values obtained from North American, European, or Australian populations, despite substantial differences in anthropometric characteristics, environmental conditions, cultural habits, and training practices across geographic regions.

The absence of locally derived reference values represents an important limitation in clinical and sports biomechanics because movement patterns are influenced by biological and environmental factors. Consequently, the establishment of reference values specific to healthy recreational runners from Barranquilla provides a more appropriate framework for biomechanical interpretation than the exclusive use of international datasets.

The percentile-based approach adopted in the present study allows clinicians and sports professionals to classify biomechanical variables according to locally derived reference intervals. From a practical perspective, values located outside the interpercentile range (P10–P90) may indicate atypical movement strategies that warrant additional biomechanical assessment or clinical monitoring.

Regarding the age distribution of our sample, although our inclusion criteria theoretically allowed the screening of individuals up to 65 years of age to ensure broad community reach, our final analyzed cohort was concentrated between 20 and 47 years. Biomechanically, this specific age range is highly appropriate for establishing baseline reference values, as it represents a mature locomotor profile while avoiding the confounding effects of age-related biomechanical declines—such as reduced preferred running speed, shorter stride lengths, and longer stance times—that are commonly observed in runners older than 50 years [3]. Consequently, this age range ensures that our reference percentiles (P10–P90) reflect a stable and representative baseline of young and middle-aged healthy recreational runners, free from age-associated degenerative locomotion adaptations.

### 4.2. Comparison with International Reference Databases

The comparison presented in Section 3.5 indicates that healthy adult recreational runners from Barranquilla display a spatiotemporal signature that departs systematically from the largest available European reference dataset [17]: a shorter flight time, a markedly lower vertical excursion of the centre of mass and a higher cadence. The interpretive value of this observation, however, does not lie in the raw group contrast, which is confounded by well-documented determinants of running mechanics, but in the fact that the three differences survive full adjustment for those determinants.

When each participant’s expected value was computed from the reference regression equations using their own sex, age, body mass, stature and treadmill speed, the observed values still fell 6.6% below prediction for flight time, 19.0% below for vertical oscillation and 3.1% above for cadence, with moderate to large paired effect sizes (dz = −1.44, −1.11 and +0.67, respectively). This result carries a consequence that qualifies the interpretation offered in earlier versions of this manuscript: the anthropometric profile of the cohort, although clearly distinct (mean stature 167.8 cm and mean body mass 69.2 kg against 174 cm and 73.1 kg in the reference sample), is by itself insufficient to account for the divergence. The reference equations already incorporate stature and body mass, and once these are held constant, the gap is essentially preserved. The shorter-leg/higher-step-frequency mechanism proposed by Patoz et al. [14] therefore remains a plausible contributory pathway, but it cannot be advanced as the principal explanation.

The same reasoning resolves the potential confounding by age. The present cohort is on average 7.5 years younger than the reference sample (32.7 ± 7.4 vs. 40.2 ± 10.0 years), and age is a significant determinant of all three parameters in the reference model. Critically, its coefficients act in the direction opposite to the observed differences: being younger predicts a longer flight time (−0.765 ms per year), a greater vertical oscillation (−0.275 mm per year) and a lower cadence (+0.193 steps/min per year). Quantitatively, the age gap would be expected to produce a flight time 5.8 ms longer, a vertical oscillation 2.1 mm greater and a cadence 1.5 steps/min lower than the reference sample, whereas the observed values are 28.7 ms shorter, 14.6 mm lower and 6.9 steps/min higher. The age difference between cohorts therefore attenuates rather than generates the observed pattern, and the true population divergence is, if anything, underestimated by the unadjusted comparison.

Mechanically, the three differences are not independent findings but a single coherent adaptation. Cadence, flight time and vertical displacement of the centre of mass are linked by the ballistic constraint of the aerial phase: for a given running velocity, a higher step frequency shortens the time available for the flight phase, and because the vertical rise in the centre of mass scales with the square of flight time, even modest reductions in aerial duration translate into disproportionately smaller vertical excursions. The observed triad—higher cadence, shorter flight time, lower vertical oscillation, accompanied by a 3.8% shorter stride than predicted—thus describes a flatter, more ground-integrated stride executed at a comparatively higher frequency. From a loading perspective, this strategy is not neutral: reduced vertical excursion of the centre of mass lowers the vertical impulse that must be absorbed at landing and is generally associated with lower peak vertical loading, which is of clinical interest given the association between loading magnitude and running-related injury in recreational populations [4,8].

Three methodological caveats must temper this interpretation before a population-level conclusion is drawn. First, the two datasets do not measure the target variables with the same instrument: the reference values were derived from ground reaction forces recorded on an instrumented treadmill, with vertical oscillation obtained by double integration of the vertical acceleration signal, whereas the present values were reconstructed from optical marker trajectories through a multi-segment centre-of-mass model. These two approaches are known to yield systematically different estimates of centre-of-mass excursion, and this instrumental non-equivalence is the most likely partial contributor to the comparatively large discrepancy observed for vertical oscillation (19.0%) relative to flight time (6.6%) and cadence (3.1%), the latter two being event-based measures far less sensitive to the reconstruction method. Second, the reference cohort ran at its self-selected preferred speed, whereas the present cohort ran at four externally imposed speeds; although the adjusted analysis controls for absolute velocity, it cannot control for the difference between self-selected and imposed pacing, which constrains stride regulation. Third, footwear was standardized in the reference study and habitual in the present study, and shoe cushioning and longitudinal bending stiffness are known modifiers of the very variables compared here. Consequently, while the regression-based covariate adjustment serves as a sensitivity check for biological confounders, the absolute differences reported in Table 3 must be interpreted as joint products of population characteristics and these unavoidable methodological discrepancies.

Taken together, the evidence supports a moderate and appropriately qualified conclusion. A genuine spatiotemporal divergence between this regional cohort and the European reference population is present and survives adjustment for sex, age, stature, body mass and running speed; it is mechanically coherent; and it is compatible with the proposition, advanced on ethnographic grounds by Wallace et al. [13], that running technique is shaped by sociocultural running habits and training environments in addition to morphology. It is not, however, attributable to anthropometry alone, and part of its magnitude—particularly for vertical oscillation—is likely to be methodological rather than biological. The practical implication is accordingly specific rather than general: applying European reference limits to runners from Barranquilla would misclassify a non-trivial proportion of healthy individuals for these parameters, which justifies locally derived reference intervals, while a definitive attribution of the residual difference to population factors will require a study in which both cohorts are measured with the same instrumentation and pacing protocol.

### 4.3. Clinical and Practical Implications

The reference values established in this study have important implications for clinical practice, sports performance, and biomedical engineering. By providing population-specific reference intervals for healthy recreational runners from Barranquilla, this study offers an objective framework for interpreting running biomechanics within a regional context.

From a clinical perspective, these reference data may improve biomechanical screening by facilitating the identification of atypical movement patterns associated with injury risk or functional impairment. Without population-specific references, clinicians in Latin America risk misdiagnosing healthy local variations as clinical anomalies. For instance, evaluating a runner from Barranquilla using European standards would incorrectly flag their lower vertical oscillation (60 mm) or shorter flight time (0.42 s) as a pathological deficit or ‘suboptimal’ technique, when in reality it represents a highly stable and normal regional running style falling comfortably within our established P10–P90 reference intervals. Conversely, identifying a local runner whose vertical oscillation exceeds 80 mm (above our P90) allows clinicians to detect true mechanical inefficiency and increased joint loading, which is a known risk factor for patellofemoral pain syndrome (PFPS) and tibial stress fractures. Consequently, these speed- and sex-specific percentile ranges offer a preliminary, regionally derived clinical baseline for injury prevention screening, personalized rehabilitation monitoring, and objective criteria for return-to-sport decision-making. Crucially, these applications are strictly constrained to motorized treadmill evaluations at matched velocities, as they cannot be directly extrapolated to overground or self-paced running without introducing significant kinematic bias.

In sports science, the proposed reference intervals may assist coaches and sports professionals in evaluating running technique, monitoring training adaptations, and optimizing performance through individualized biomechanical assessments. The use of speed- and sex-specific reference values enables more precise interpretation of running mechanics and may contribute to evidence-based training strategies.

To make this framework operational, we propose the following sequence of use. First, the runner should be assessed under conditions matching those in which the reference values were generated: motorized treadmill, one of the four evaluated speeds, and the runner’s habitual footwear. Second, the measured value should be read against the sex- and speed-specific P10–P90 interval in Table 2 and, simultaneously, against the corresponding bootstrap confidence interval in Supplementary Table S2; a value lying outside P10–P90 but within the confidence band of that limit should be treated as indeterminate rather than atypical. Third, an isolated deviation in a single parameter has limited clinical meaning, whereas a coherent constellation—for example a simultaneously low cadence, long flight time and high vertical oscillation—describes a mechanically interpretable overstriding pattern and provides a more defensible target for intervention. Fourth, and consistent with the reference literature [17], these intervals describe what is typical in a healthy regional population, not what is optimal; they should not be used as performance targets, and gait retraining should be considered only when an atypical value is clinically correlated with the presenting complaint.

Beyond clinical and sports applications, the resulting database represents a valuable resource for biomedical engineering. Population-specific biomechanical data may support the development of wearable monitoring systems, digital biomechanical twins, machine-learning algorithms, and predictive models for injury prevention and performance optimization. Consequently, the present study contributes not only to the understanding of running biomechanics but also to the advancement of digital health technologies and personalized movement analysis.

### 4.4. Future Directions

Future research should expand the reference database by incorporating kinetic variables, muscle activation patterns, and wearable sensor technologies. The integration of kinematic, kinetic, and electromyographic information may facilitate the development of comprehensive biomechanical models capable of improving injury prediction and personalized intervention strategies.

Additionally, comparative studies involving different Colombian regions and Latin American populations may help determine whether regional biomechanical differences exist and whether population-specific reference values should be routinely incorporated into clinical practice.

Finally, a reference database is a living instrument rather than a fixed reference, and explicit governance rules are required as it grows. We recommend that these reference values be revised whenever any of three conditions is met: (i) the sample within a given sex × speed stratum reaches *n* ≥ 30, the point at which the bootstrap confidence intervals reported in Supplementary Table S2 narrow sufficiently for P10 and P90 to be estimated with acceptable precision; (ii) the cumulative sample exceeds 100 participants, which would permit the current stratified percentile approach to be replaced by reference regression equations adjusted for sex, age, stature, body mass and speed, as implemented in larger cohorts [17]; or (iii) the recruitment base is broadened beyond the present convenience sample, for example, through multi-centre collaboration across Colombian regions. Until the second condition is met, the values reported here should be regarded as preliminary reference intervals for a defined regional population, and each update should report the version of the database and the stratum sizes on which it is based so that clinical decisions remain traceable to a specific data release.

### 4.5. Study Limitations

The primary methodological limitation of this study lies in the fact that biomechanical data collection was conducted exclusively on a motorized treadmill under controlled laboratory conditions. Although treadmill running is the standard approach for capturing multiple consecutive 3D kinematic cycles at standardized speeds (7, 9, 11, and 13 km/h), the comparative scientific literature has demonstrated subtle, yet relevant kinematic differences compared with overground running [25,26,27]. Specifically, treadmill running is typically characterized by a flatter foot angle at initial contact (reduced ankle dorsiflexion), a slight increase in knee flexion range of motion during stance, and mechanical adaptations arising from the continuous motion of the treadmill belt [25,26].

Furthermore, the absence of aerodynamic drag and the static visual scene in the laboratory environment influence vertical center-of-mass modulation, explaining why the vertical oscillation values observed in this cohort (0.06 ± 0.01 m) are slightly lower than those reported on rigid overground surfaces [17,25,28]. Additionally, the fixed speed imposed by the treadmill motor induces more constrained and regular muscle activation and kinematic patterns than those observed during self-propelled locomotion overground [29]. Therefore, it is explicitly declared that the reported reference values (10th–90th percentiles) apply specifically to motorized treadmill running and may require empirical validation and adjustment for extrapolation to overground, asphalt, or track conditions.

Finally, although the sample size (*n* = 25 participants; 98 valid running reports) is methodologically robust and consistent with international recommendations for 3D kinematic laboratory studies [15], the cohort was derived from a convenience sample of healthy recreational runners from Barranquilla. Although we mitigated selection bias by actively recruiting from local running clubs and social networks, we acknowledge that a single-center sample may not fully capture the complete socioeconomic, ethnic, or training diversity of all recreational runners within the city. Additionally, while letting participants run in their habitual footwear preserves ecological validity and reflects their natural biomechanical state, variations in shoe design (e.g., cushioning and drop) could influence individual kinematic signatures.

A related limitation concerns the statistical precision of the reference limits themselves. With 11 to 13 valid observations per sex × speed stratum, the extreme percentiles are estimated with appreciable uncertainty: the bootstrap 95% confidence intervals reported in Supplementary Table S2 are, for several variables, of a width comparable to the P10–P90 interval itself. The reference limits should therefore be used as an orientation for screening rather than as sharp diagnostic thresholds, and an individual value falling marginally outside them should not be interpreted as atypical without clinical corroboration. For the same reason, the inferential findings should be read with attention to the multiplicity adjustment: after Holm correction, the speed effect on the initial contact angle and on vertical oscillation no longer reached the conventional threshold in the factorial model, although both were recovered in the mixed-effects model that accounts for the repeated-measures structure. Conversely, the sex effects were attenuated once within-participant dependence was modelled (ICC = 0.71–0.88) and remained robust only for flight time and vertical leg stiffness. Sex-stratified reference values are retained here for clinical usability, but the magnitude of the sex effect for the remaining variables should be regarded as provisional pending a larger cohort.

Additionally, we acknowledge that footwear was not standardized across participants. Although requiring participants to run in their habitual training shoes (as detailed in Section 2.7) preserves ecological validity and reflects their natural biomechanical state, different shoe designs—varying in cushioning, longitudinal bending stiffness, and heel-to-toe drop—can significantly influence running kinematics and center-of-mass dynamics. The lack of strict control over footwear characteristics must therefore be recognized as a limitation.

The estimation of vertical leg stiffness warrants a specific caveat. Because no force platform was integrated into the treadmill, stiffness was not measured but modelled from the sine-wave mass-spring approximation of Morin et al. [20], which reconstructs the peak vertical ground reaction force from body mass, contact time and flight time under the assumption that the vertical force–time curve is adequately described by a half-sine wave and that leg compression can be derived from leg length and horizontal velocity. These assumptions hold reasonably well at endurance speeds in rearfoot strikers but degrade at the extremes of contact time and in runners with markedly non-sinusoidal loading profiles, and the method does not capture the impact-peak component of the vertical force. The stiffness values reported here should consequently be interpreted as model-derived estimates suitable for within-cohort comparison rather than as absolute measurements, and they are not directly comparable with force-plate-derived stiffness reported elsewhere, which is why vertical leg stiffness was excluded from the international comparison in Section 3.5. Accordingly, these model-derived values should be validated against direct force-plate measurements before they are adopted for clinical diagnostics or cross-study comparisons.

Furthermore, we acknowledge that direct test–retest reliability, Standard Error of Measurement (SEM), and Minimal Detectable Change (MDC) were not calculated within our specific regional cohort. To mitigate this and minimize marker-placement error, all reflective markers were attached by the same highly trained investigator, effectively eliminating inter-rater variability. Furthermore, the laboratory utilized a high-precision gold-standard 3D motion capture system calibrated daily, achieving a spatial resolution of 0.1 mm. The extensive biomechanical literature using identical optical tracking technology and Modified Helen Hayes protocols has consistently demonstrated excellent test–retest reliability (ICC > 0.85) and low measurement errors for spatiotemporal and vertical kinematic variables. Nonetheless, future studies establishing regional reference databases should consider incorporating multi-session test–retest designs to establish cohort-specific SEM and MDC values.

Lastly, training-load characteristics beyond the eligibility criteria—weekly running volume, years of running experience, session frequency and competitive history—were not systematically recorded. Because running experience and weekly volume are recognised determinants of several spatiotemporal variables, their absence limits the degree to which the present cohort can be positioned within the broader recreational running population, and future data collection waves for this database will incorporate a standardised training-history questionnaire.

## 5. Conclusions

This established preliminary spatiotemporal and selected kinematic reference values for a specific cohort of healthy adult recreational runners from Barranquilla using three-dimensional motion capture technology. To the authors’ knowledge, this is the first investigation to provide preliminary regional baseline values for running biomechanics in this specific group, which should be interpreted as an initial cohort reference rather than a generalized, population-wide norm.

The development of percentile-based reference intervals constitutes the principal contribution of this research. By defining reference ranges between the 10th and 90th percentiles for temporal, spatial, and kinematic variables, the study provides an objective framework for biomechanical assessment in clinical, sports, and research settings. These reference values allow practitioners to compare individual running patterns against this initial regional cohort of healthy recreational runners—acknowledging that further multi-center studies are required to establish fully representative national or regional standards and facilitate the identification of biomechanical deviations that may be associated with injury risk, functional limitations, or altered movement strategies.

From a clinical perspective, the generated reference values may support biomechanical screening, rehabilitation monitoring, return-to-sport decision-making, and injury-prevention programs. The availability of locally derived reference data is particularly relevant because environmental, anthropometric, and cultural factors may influence running mechanics and limit the applicability of international reference datasets.

From a sports science and biomedical engineering perspective, the resulting database provides a foundation for future developments involving wearable technologies, biomechanical simulations, machine-learning applications, and digital health solutions aimed at improving running performance and reducing injury risk.

Future investigations should expand these findings by incorporating larger multi-center cohorts that include diverse regions of Colombia, overground running assessments, kinetic analyses, and electromyographic measurements. The integration of kinematic, kinetic, and neuromuscular information may contribute to the development of comprehensive biomechanical models capable of supporting personalized interventions and predictive approaches in sports medicine and rehabilitation.

Overall, the present study contributes to reducing an important knowledge gap in Latin American biomechanics and provides the first reference running kinematic database specifically developed for healthy adult runners from Barranquilla.

## Figures and Tables

**Figure 2:**
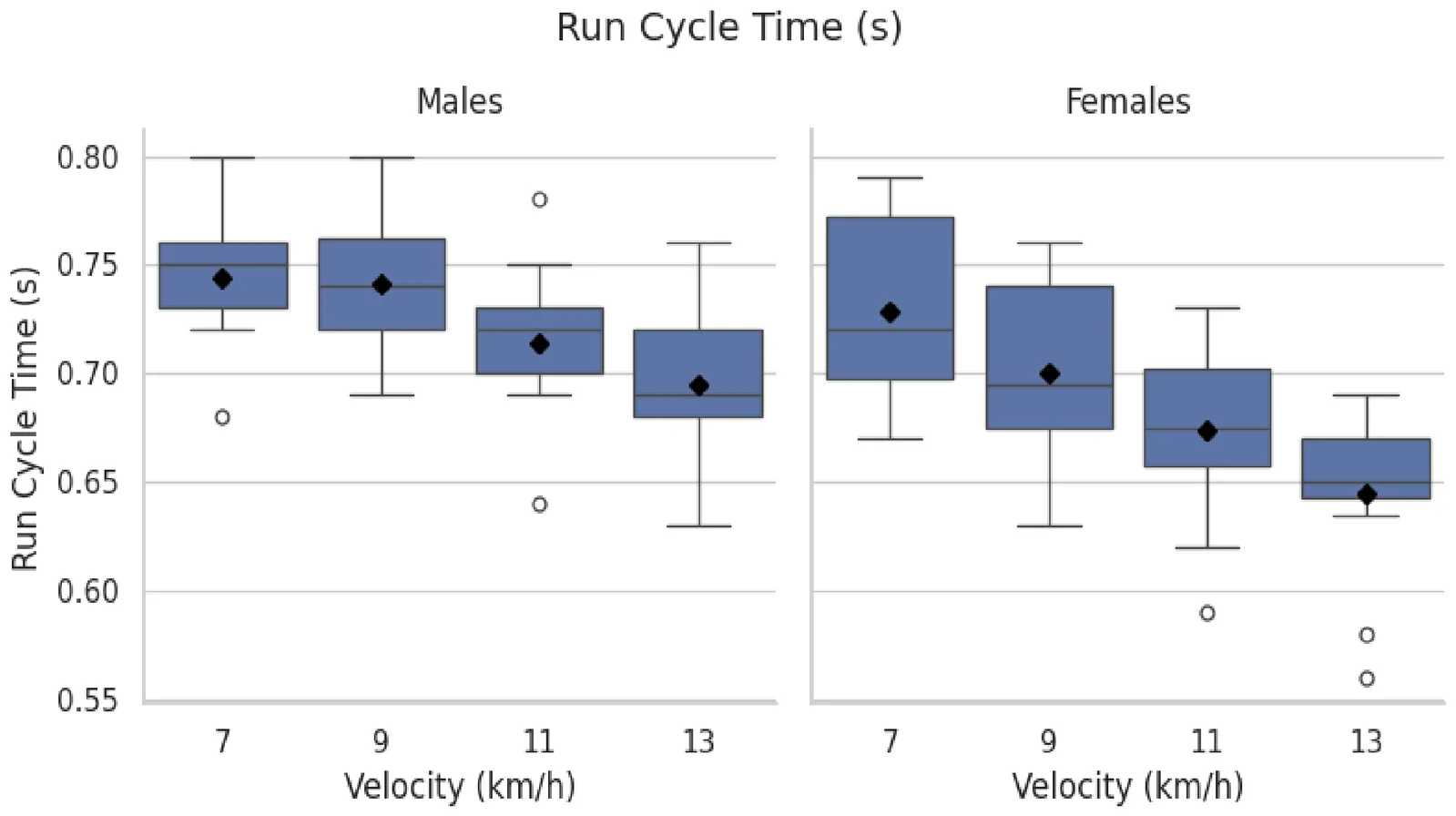
Boxplots of running cycle time according to sex and running speed.

**Figure 3:**
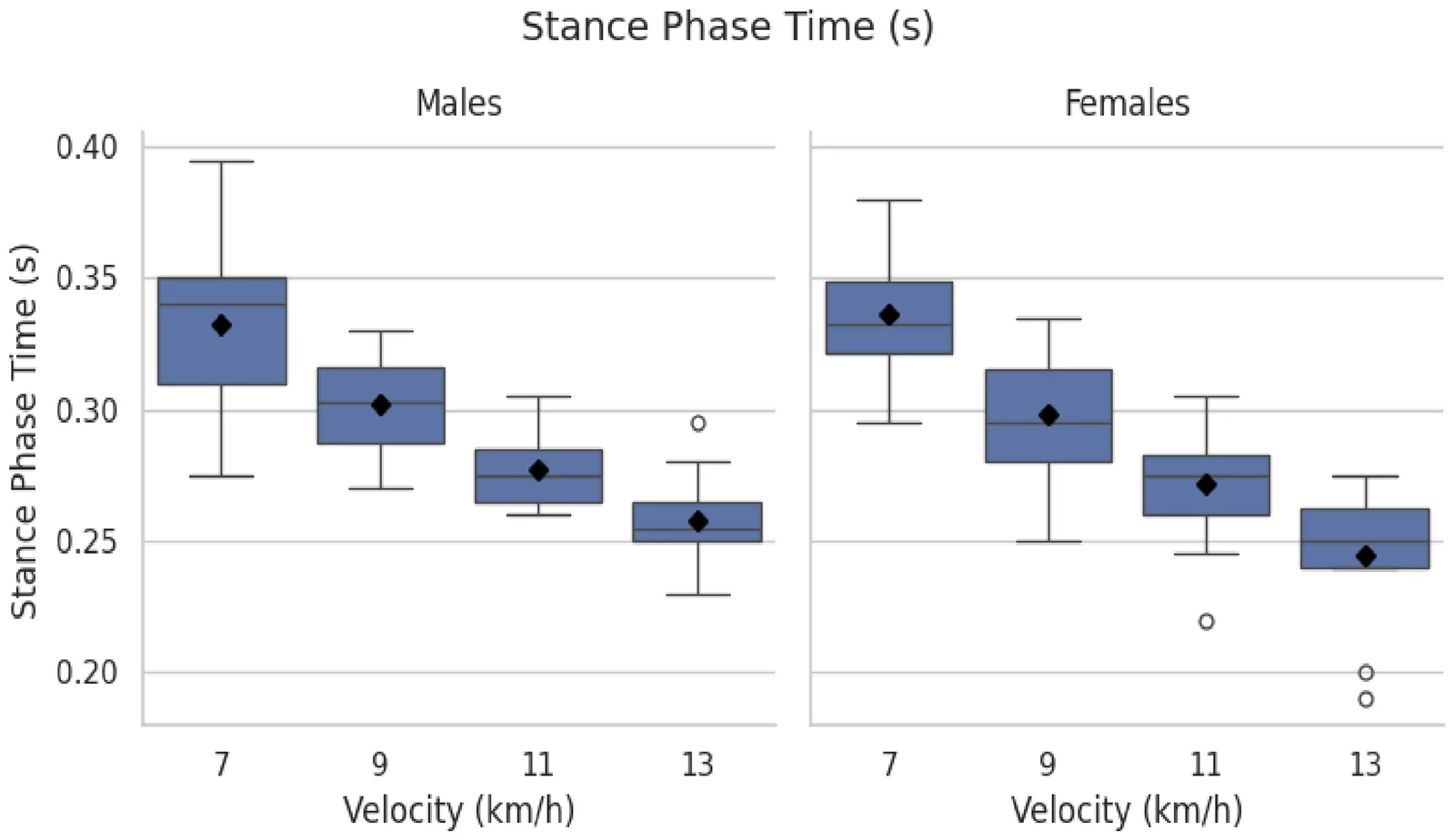
Boxplots of stance phase time according to sex and running speed.

**Figure 4:**
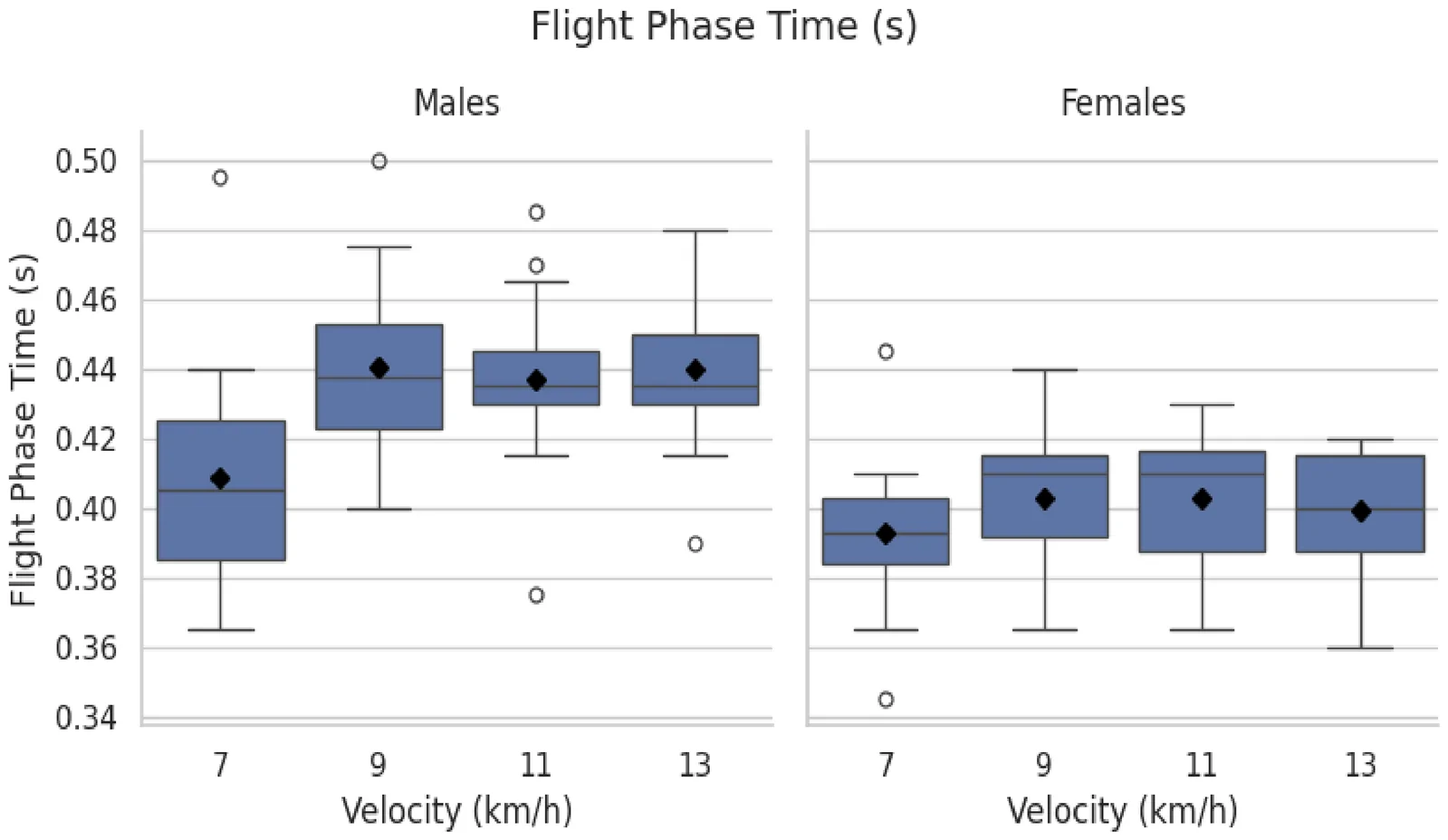
Boxplots of flight phase time according to sex and running speed.

**Figure 5:**
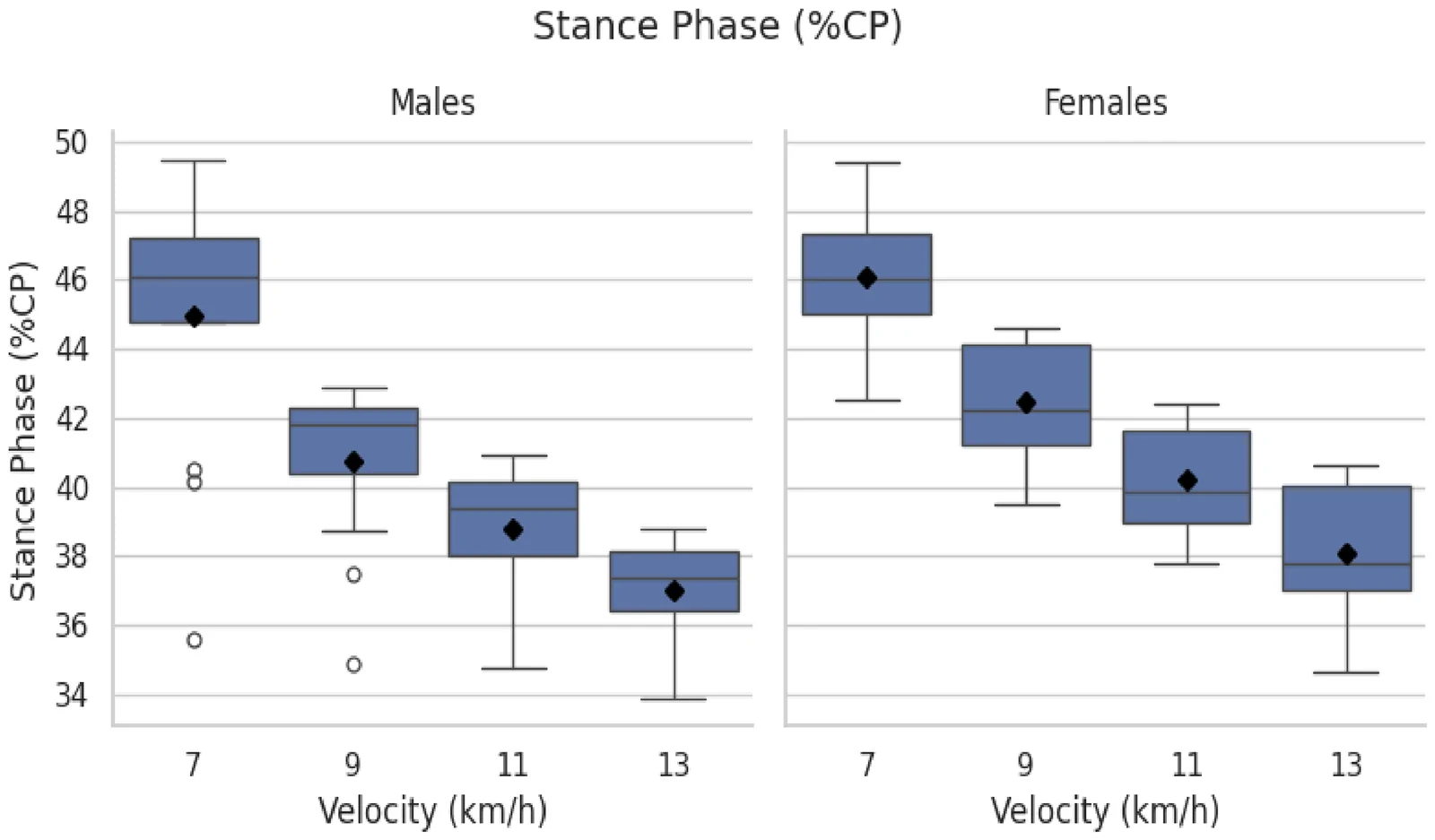
Boxplots of stance phase according to sex and running speed.

**Figure 6:**
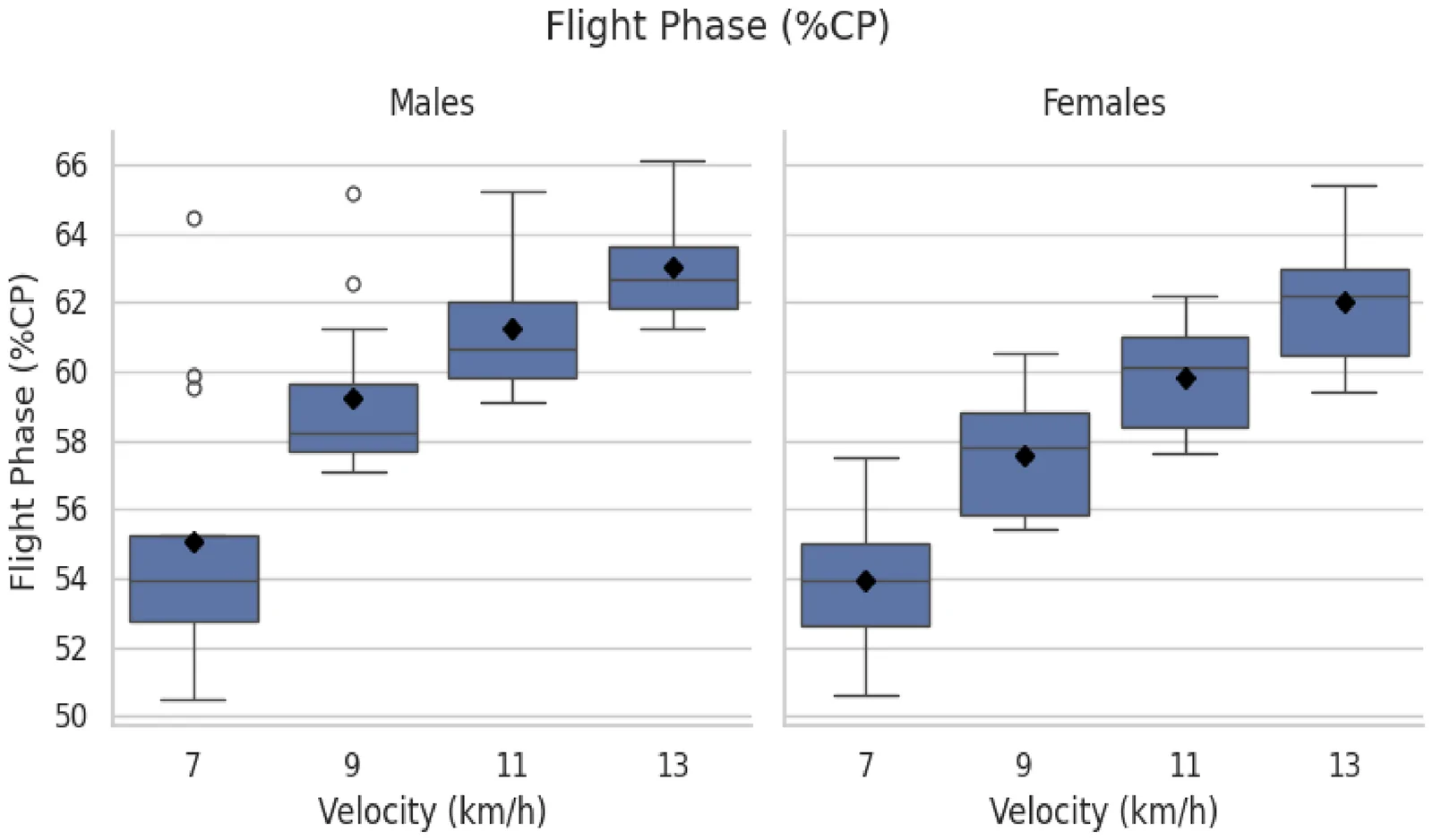
Boxplots of flight phase percentage according to sex and running speed.

**Figure 7:**
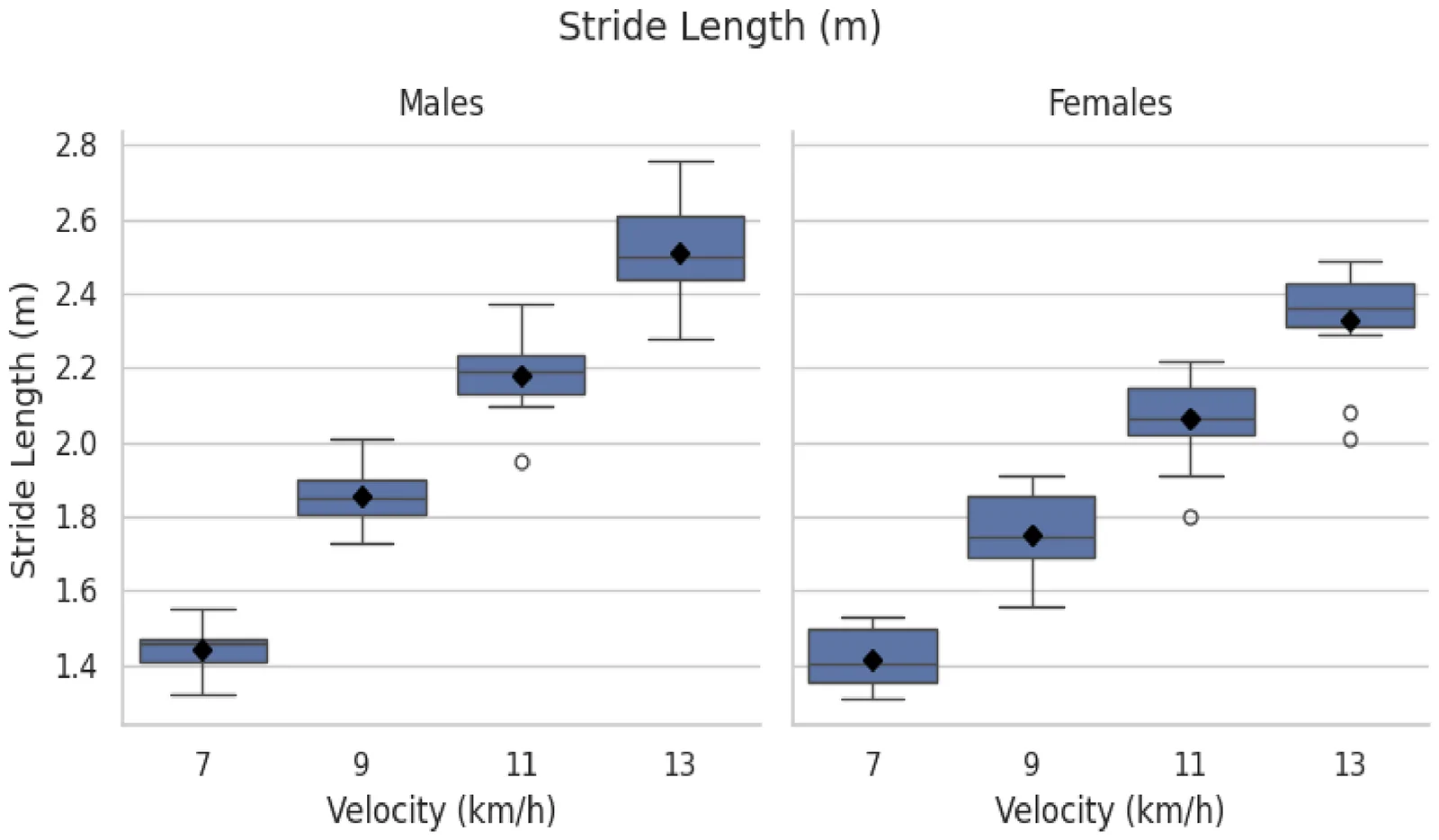
Boxplots of stride length according to sex and running speed.

**Figure 8:**
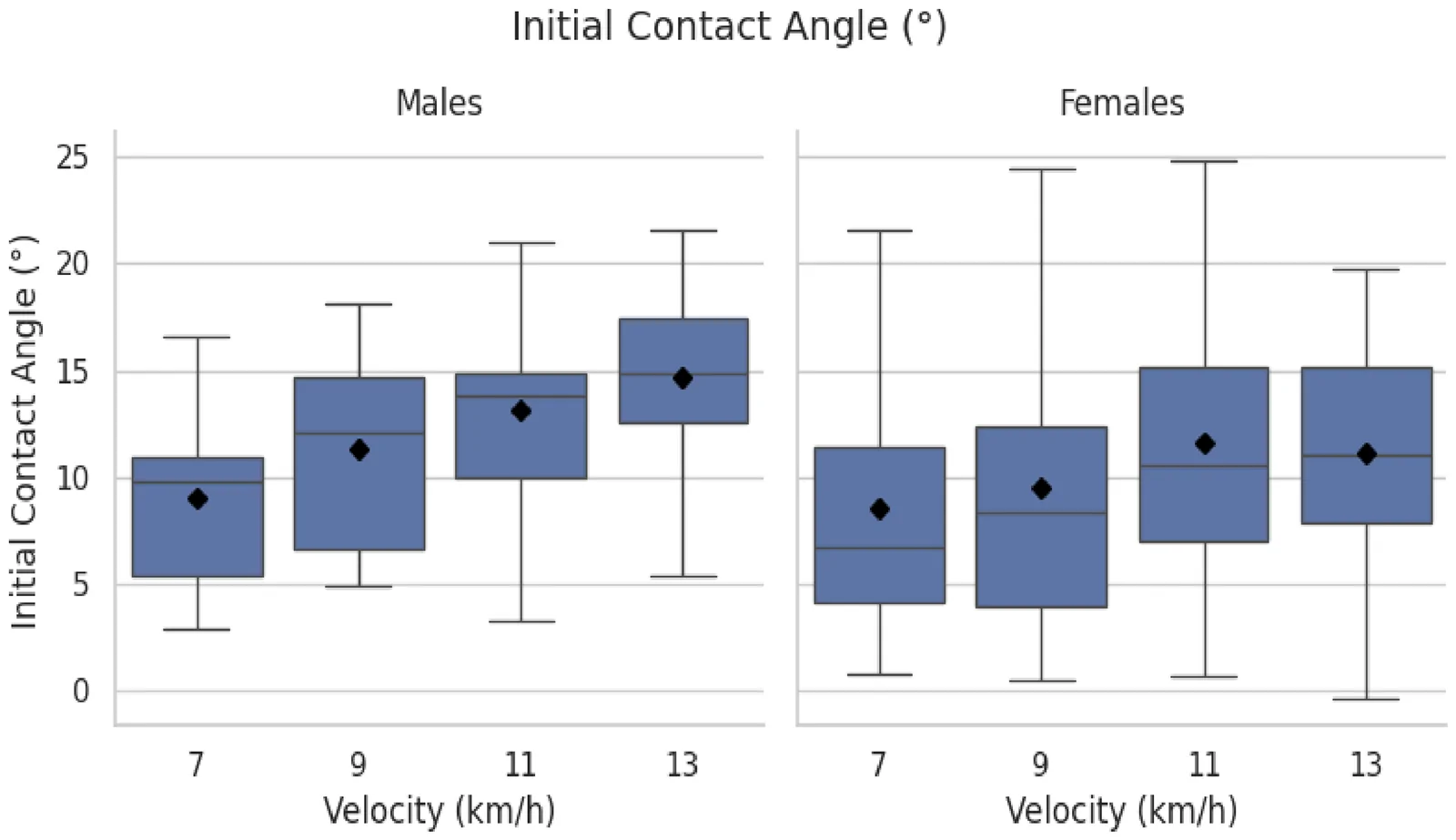
Boxplots of initial contact angle according to sex and running speed. The angle represents the sagittal segmental orientation of the foot relative to the horizontal treadmill surface at initial contact. Positive values indicate a dorsiflexed foot position (rearfoot strike pattern), while negative values represent a plantarflexed foot position (midfoot/forefoot strike pattern).

**Figure 9:**
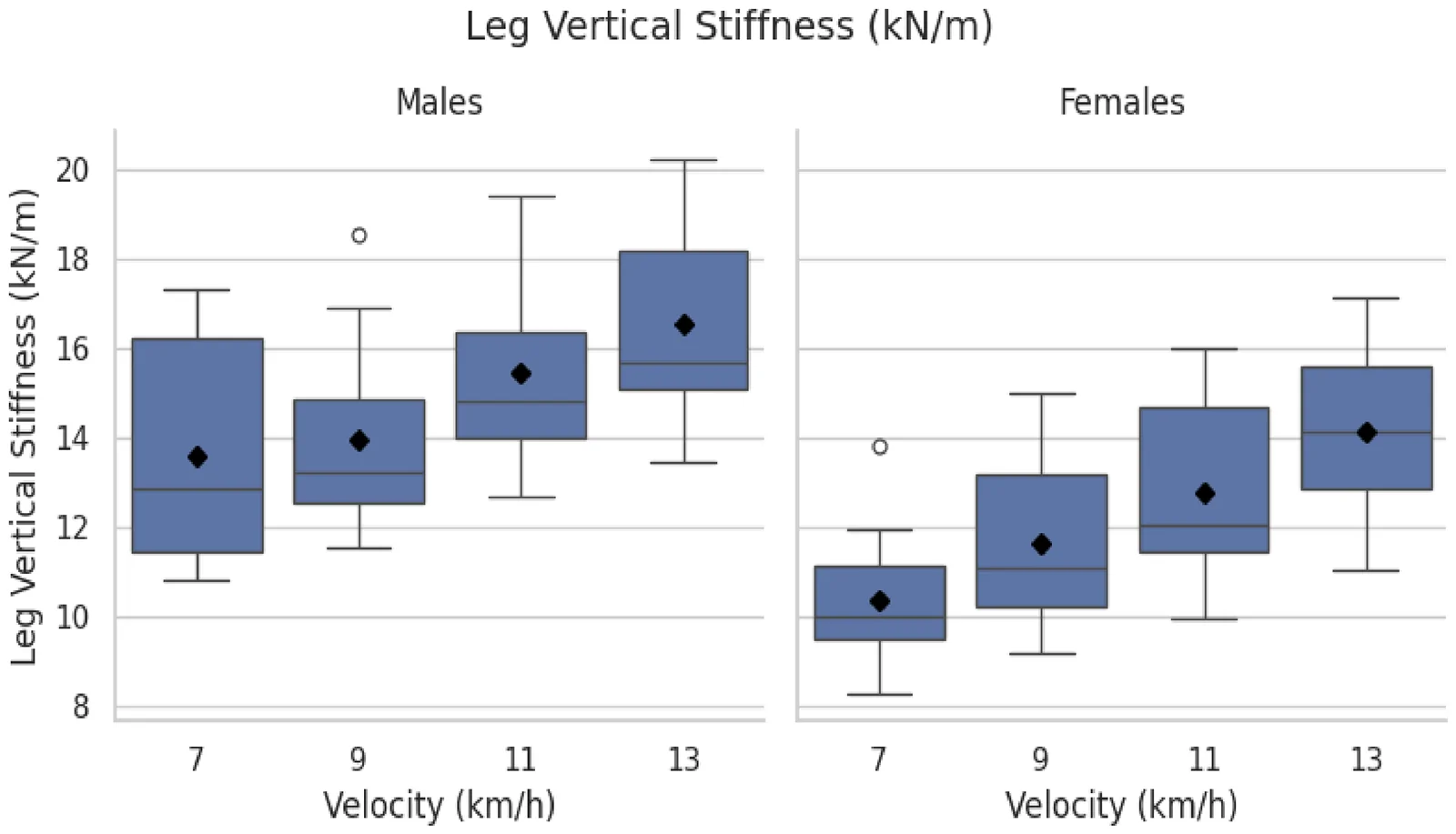
Boxplots of leg vertical stiffness according to sex and running speed.

**Figure 10:**
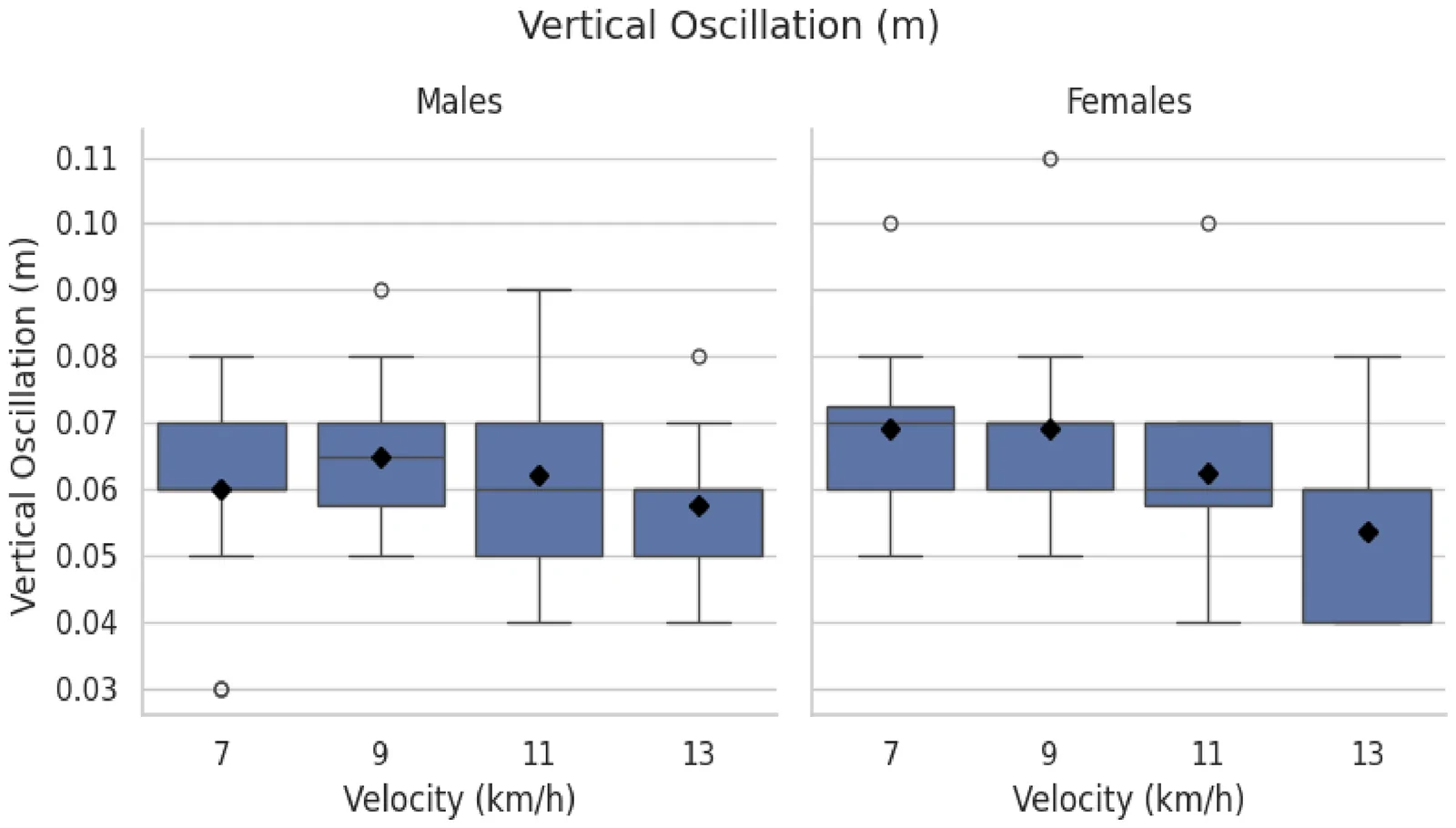
Boxplots of vertical oscillation according to sex and running speed.

**Figure 11:**
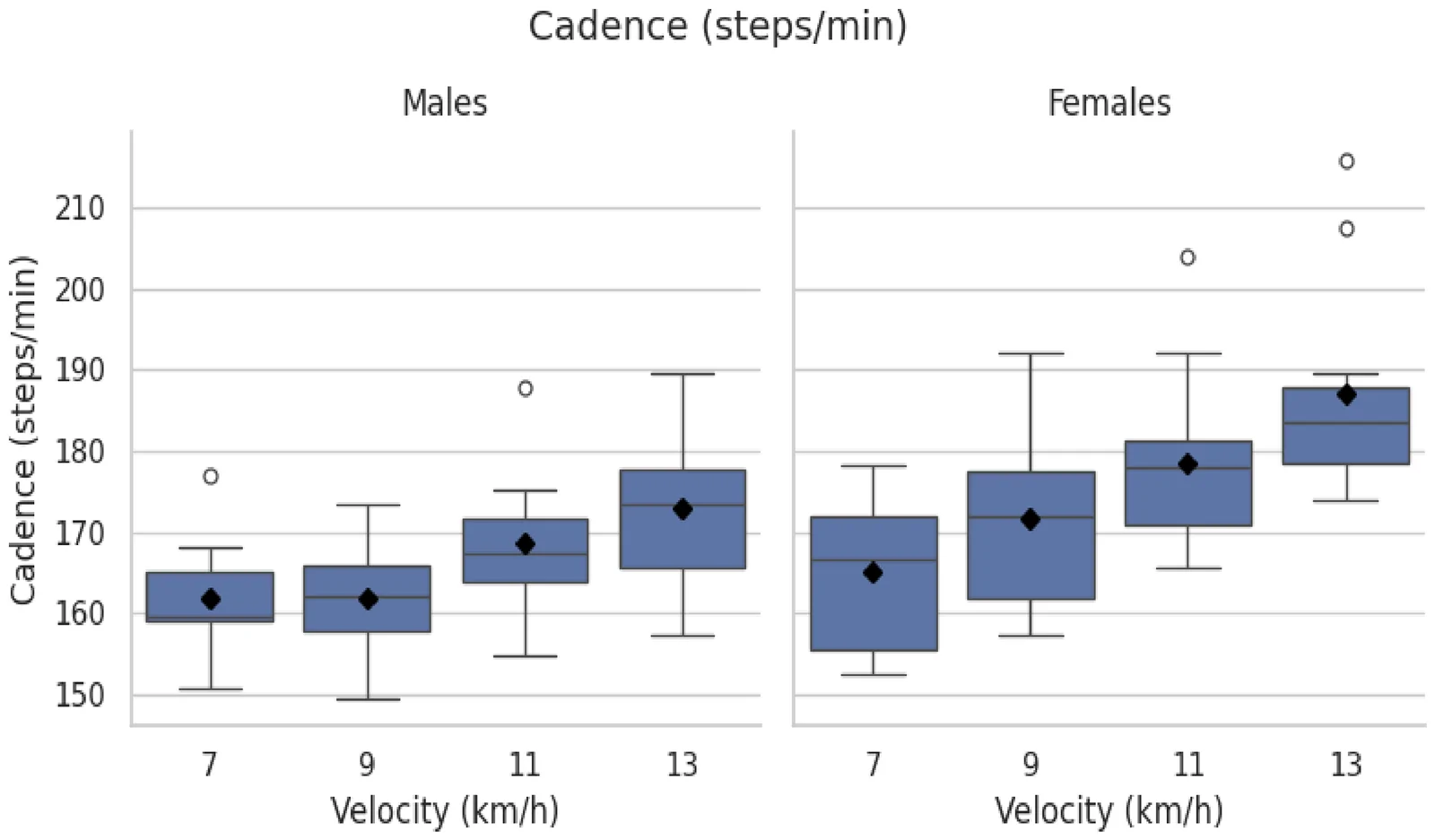
Boxplots of cadence according to sex and running speed.

**Table 3:** Comparison of selected spatiotemporal parameters between the Barranquilla cohort and the European reference sample of Malisoux et al. [17]. (**A**) unadjusted comparison of group means; (**B**) comparison of observed values against values individually predicted by the reference regression equations.

Parameter	Barranquilla (*n* = 25)	Reference (*n* = 860)	Mean Difference (95% CI)	*t* (df)	*p*	Effect Size
(**A**) Unadjusted comparison against the reference cohort (Welch’s *t*-test)						
Flight time (s)	0.415 ± 0.029	0.444 ± 0.038	−0.029 (−0.041 to −0.017)	−4.86 (26.5)	<0.001	g = −0.76
Vertical oscillation (m)	0.062 ± 0.013	0.077 ± 0.012	−0.015 (−0.020 to −0.009)	−5.72 (25.3)	<0.001	g = −1.22
Cadence (steps/min)	170.9 ± 9.9	164.0 ± 9.0	+6.89 (+2.75 to +11.02)	3.43 (25.2)	0.002	g = +0.76
(**B**) Adjusted comparison: observed values vs. values individually predicted by the reference regression equations (column 3 = predicted value, *n* = 25; paired *t*-test)						
Flight time (s)	0.415	0.444	−0.029 (−0.038 to −0.021)	−7.22 (24)	<0.001	dz = −1.44
Vertical oscillation (m)	0.062	0.077	−0.015 (−0.020 to −0.009)	−5.56 (24)	<0.001	dz = −1.11
Cadence (steps/min)	170.9	165.7	+5.15 (+1.96 to +8.35)	3.33 (24)	0.003	dz = +0.67
Stride length (m)	1.939	2.016	−0.077 (−0.112 to −0.042)	−4.58 (24)	<0.001	dz = −0.92

Note. Values are mean ± SD. Reference data correspond to healthy adult recreational runners tested at their preferred running speed on an instrumented treadmill [17]. In Panel A, each participant contributed one value per parameter (mean across completed speed conditions) to avoid pseudo-replication. In Panel B, the predicted value was computed for every observation from the reference regression equations using that participant’s sex, age, body mass, height and treadmill speed, and then averaged per participant. CI, confidence interval; g, Hedges’ g; dz, Cohen’s dz for paired samples.

## Data Availability

The datasets generated and analyzed during the current study are available from the corresponding author upon reasonable request. The complete anonymised participant-level dataset supporting all analyses is additionally provided as Supplementary Table S1.

## Supplementary Materials

The following supporting information can be downloaded at: https://www.mdpi.com/article/10.3390/jfmk11030350/s1, Table S1: Descriptive statistics of the participants for the full sample and sensitivity sample. Values are presented as mean ± SD, except for age, which is presented as mean [range], and sex, which is presented as percentage. The sensitivity sample excludes the two participants with an incomplete four-speed series; Table S2: Percentile estimates of running biomechanical variables for the full and sensitivity samples. Values are presented as P10, P25, P50, P75, and P90. *n* indicates the number of participants at each running speed and sex. The sensitivity sample excludes the two participants with an incomplete four-speed series.
