# Supplementary material for: Establishment of Reference Values for Selected Spatiotemporal and Kinematic Running Parameters in Healthy Adult Recreational Runners from Barranquilla, Colombia
Source: J Funct Morphol Kinesiol. 2026 Sep 3;11(3):350. doi: 10.3390/jfmk11030350 (PMC13608732; doi:10.3390/jfmk11030350)
Supplement: Supplementary file 1 [file jfmk-11-00350-s001.zip › jfmk-4432275-supplementary.pdf]

Full sample (InBody n=25; Running n=98)				Sensitivity sample (InBody n=23; Running n=92)			
**Variable**	**Total**	**Men**	**Women**	**Variable**	**Total**	**Men**	**Women**
Age, y	32.68 [20-47]	31.85 [20-41]	33.58 [21-47]	Age, y	32.17 [20-47]	31.08 [20-41]	33.36 [21-47]
Height, cm	167.76 ± 8.21	174.46 ± 3.99	160.50 ± 4.36	Height, cm	167.61 ± 8.52	174.67 ± 4.10	159.91 ± 4.04
Body mass, kg	69.15 ± 9.31	76.39 ± 5.36	61.30 ± 5.36	Body mass, kg	68.65 ± 9.03	75.65 ± 4.85	61.02 ± 5.52
BMI, kg/m2	24.52 ± 2.27	25.15 ± 2.20	23.83 ± 2.23	BMI, kg/m2	24.39 ± 2.16	24.84 ± 2.00	23.90 ± 2.32
PBF, Percent Body Fat, %	23.68 ± 8.35	18.23 ± 6.11	29.58 ± 6.18	PBF, Percent Body Fat, %	23.54 ± 8.68	17.84 ± 6.21	29.76 ± 6.45
SMM, Skeletal Muscle Mass, kg	29.84 ± 6.48	35.59 ± 2.16	23.60 ± 2.24	SMM, Skeletal Muscle Mass, kg	29.67 ± 6.50	35.42 ± 2.15	23.41 ± 2.24
Men, %	52.0			Men, %	52.2		
Women, %	48.0			Women, %	47.8		
Run cycle time, s	0.71 ± 0.05	0.72 ± 0.04	0.69 ± 0.05	Run cycle time, s	0.71 ± 0.05	0.73 ± 0.03	0.69 ± 0.05
Stance phase time, s	0.29 ± 0.04	0.29 ± 0.04	0.29 ± 0.04	Stance phase time, s	0.29 ± 0.04	0.29 ± 0.04	0.29 ± 0.04
Flight phase time, s	0.42 ± 0.03	0.43 ± 0.03	0.40 ± 0.02	Flight phase time, s	0.42 ± 0.03	0.43 ± 0.03	0.40 ± 0.02
Stance phase, %CP	41.04 ± 3.79	40.36 ± 3.97	41.78 ± 3.48	Stance phase, %CP	40.93 ± 3.81	40.28 ± 4.00	41.65 ± 3.50
Flight phase, %CP	58.97 ± 3.80	59.64 ± 3.97	58.25 ± 3.49	Flight phase, %CP	59.08 ± 3.81	59.72 ± 4.00	58.38 ± 3.51
Stride length, m	1.94 ± 0.39	2.00 ± 0.41	1.88 ± 0.36	Stride length, m	1.95 ± 0.39	2.01 ± 0.41	1.89 ± 0.36
Initial contact angle, °	11.18 ± 5.88	12.08 ± 4.83	10.20 ± 6.75	Initial contact angle, °	10.89 ± 5.91	11.96 ± 4.91	9.71 ± 6.70
Leg vertical stiffness, kN/m	13.61 ± 2.73	14.91 ± 2.47	12.20 ± 2.28	Leg vertical stiffness, kN/m	13.47 ± 2.64	14.69 ± 2.33	12.14 ± 2.32
Vertical Oscillation, m	0.06 ± 0.01	0.06 ± 0.01	0.06 ± 0.02	Vertical Oscillation, m	0.06 ± 0.01	0.06 ± 0.01	0.06 ± 0.02
Cadence, steps/min	170.72 ± 12.06	166.42 ± 8.71	175.39 ± 13.46	Cadence, steps/min	170.20 ± 12.07	165.28 ± 7.48	175.57 ± 13.80

Table S1. Descriptive statistics of the participants for the full sample and sensitivity sample. Values are presented as mean ± SD, except for age, which is presented as mean [range], and sex, which is presented as percentage. The sensitivity sample excludes the two participants with an incomplete four-speed series.

Full sample													
Variable	Velocity	Males						Females					
		n	P10	P25	P50	P75	P90	n	P10	P25	P50	P75	P90
Run Cycle Time (s)	7	13	0.72	0.73	0.75	0.76	0.77	12	0.68	0.70	0.72	0.77	0.78
	9	12	0.71	0.72	0.74	0.76	0.77	12	0.65	0.68	0.70	0.74	0.75
	11	13	0.69	0.70	0.72	0.73	0.75	12	0.62	0.66	0.68	0.70	0.71
	13	13	0.66	0.68	0.69	0.72	0.73	11	0.58	0.64	0.65	0.67	0.68
Stance Phase Time (s)	7	13	0.29	0.31	0.34	0.35	0.36	12	0.31	0.32	0.33	0.35	0.38
	9	12	0.28	0.29	0.30	0.32	0.33	12	0.28	0.28	0.30	0.32	0.33
	11	13	0.26	0.26	0.28	0.29	0.29	12	0.25	0.26	0.28	0.28	0.30
	13	13	0.24	0.25	0.26	0.26	0.28	11	0.20	0.24	0.25	0.26	0.27
Flight Phase Time (s)	7	13	0.37	0.38	0.40	0.42	0.44	12	0.37	0.38	0.39	0.40	0.41
	9	12	0.41	0.42	0.44	0.45	0.47	12	0.37	0.39	0.41	0.42	0.42
	11	13	0.42	0.43	0.44	0.44	0.47	12	0.38	0.39	0.41	0.42	0.42
	13	13	0.42	0.43	0.44	0.45	0.47	11	0.38	0.39	0.40	0.42	0.42
Stance Phase (%CP)	7	13	40.21	44.76	46.08	47.24	48.96	12	43.39	45.02	46.05	47.36	48.49
	9	12	37.60	40.38	41.80	42.30	42.61	12	40.99	41.20	42.21	44.14	44.52
	11	13	35.67	38.02	39.38	40.17	40.39	12	38.60	38.97	39.86	41.63	42.25
	13	13	34.98	36.40	37.34	38.14	38.42	11	34.90	37.03	37.80	40.02	40.50
Flight Phase (%CP)	7	13	51.04	52.76	53.92	55.23	59.81	12	51.51	52.63	53.95	54.99	56.61
	9	12	57.40	57.70	58.20	59.62	62.41	12	55.48	55.86	57.79	58.80	59.02
	11	13	59.62	59.82	60.62	61.99	64.33	12	57.76	58.38	60.14	61.03	61.40
	13	13	61.58	61.86	62.64	63.60	65.03	11	59.52	60.46	62.20	62.97	65.10
Stride Length (m)	7	13	1.39	1.41	1.46	1.47	1.50	12	1.32	1.35	1.40	1.50	1.52
	9	12	1.78	1.81	1.85	1.90	1.93	12	1.62	1.69	1.74	1.85	1.88
	11	13	2.10	2.13	2.19	2.24	2.27	12	1.92	2.02	2.06	2.15	2.18
	13	13	2.39	2.44	2.50	2.61	2.63	11	2.08	2.31	2.36	2.43	2.46
Initial Contact Angle (°)	7	13	4.16	5.35	9.75	10.90	13.19	12	1.53	4.15	6.75	11.45	16.61
	9	12	5.94	6.62	12.08	14.62	17.58	12	1.55	3.91	8.32	12.41	19.50
	11	13	8.38	9.95	13.85	14.90	18.48	12	5.24	7.01	10.58	15.19	20.36
	13	13	10.11	12.55	14.85	17.40	19.99	11	4.30	7.88	11.05	15.12	19.35

Sensitivity sample													
Variable	Velocity	Males						Females					
		n	P10	P25	P50	P75	P90	n	P10	P25	P50	P75	P90
Run cycle time (s)	7	12	0.72	0.73	0.75	0.76	0.77	11	0.68	0.70	0.71	0.78	0.78
	9	12	0.71	0.72	0.74	0.76	0.77	11	0.65	0.67	0.70	0.74	0.75
	11	12	0.69	0.70	0.72	0.73	0.75	11	0.62	0.66	0.68	0.70	0.72
	13	12	0.67	0.68	0.69	0.72	0.73	11	0.58	0.64	0.65	0.67	0.68
Stance phase time (s)	7	12	0.29	0.32	0.34	0.35	0.36	11	0.31	0.32	0.33	0.35	0.38
	9	12	0.28	0.29	0.30	0.32	0.33	11	0.28	0.28	0.30	0.32	0.34
	11	12	0.26	0.27	0.28	0.29	0.29	11	0.24	0.26	0.28	0.29	0.30
	13	12	0.25	0.25	0.26	0.27	0.28	11	0.20	0.24	0.25	0.26	0.27
Flight phase time (s)	7	12	0.38	0.39	0.41	0.43	0.44	11	0.36	0.38	0.40	0.40	0.41
	9	12	0.41	0.42	0.44	0.45	0.47	11	0.37	0.39	0.41	0.42	0.42
	11	12	0.42	0.43	0.44	0.45	0.47	11	0.38	0.39	0.41	0.42	0.42
	13	12	0.42	0.43	0.44	0.46	0.47	11	0.38	0.39	0.40	0.42	0.42
Stance phase (%CP)	7	12	40.17	43.70	45.70	47.57	49.01	11	43.25	44.91	45.96	47.36	48.54
	9	12	37.60	40.38	41.80	42.30	42.61	11	40.98	41.17	41.89	44.26	44.53
	11	12	35.44	37.90	39.28	40.06	40.36	11	38.56	38.97	39.62	41.78	42.27
	13	12	34.96	36.10	37.11	38.15	38.42	11	34.90	37.03	37.80	40.02	40.50
Flight phase (%CP)	7	12	50.99	52.43	54.28	56.30	59.84	11	51.46	52.64	54.04	55.12	56.74
	9	12	57.40	57.70	58.20	59.62	62.41	11	55.47	55.74	58.11	58.83	59.04
	11	12	59.66	59.94	60.72	62.10	64.56	11	57.74	58.24	60.39	61.03	61.44
	13	12	61.58	61.85	62.88	63.92	65.05	11	59.52	60.46	62.20	62.97	65.10
Stride length (m)	7	12	1.40	1.41	1.46	1.48	1.50	11	1.32	1.35	1.39	1.50	1.52
	9	12	1.78	1.81	1.85	1.90	1.93	11	1.62	1.67	1.76	1.86	1.88
	11	12	2.12	2.14	2.19	2.24	2.28	11	1.91	2.02	2.08	2.16	2.18
	13	12	2.42	2.45	2.50	2.62	2.63	11	2.08	2.31	2.36	2.43	2.46
Initial Contact Angle (°)	7	12	4.11	5.16	9.20	11.16	13.34	11	1.30	3.95	6.10	9.48	16.25
	9	12	5.94	6.62	12.08	14.62	17.58	11	1.40	3.58	6.90	10.58	19.75
	11	12	8.32	9.69	13.90	15.72	18.51	11	5.20	6.52	8.55	14.12	20.60
	13	12	9.93	12.30	14.48	17.44	20.30	11	4.30	7.88	11.05	15.12	19.35

Full sample													
Variable	Velocity	Males						Females					
		n	P10	P25	P50	P75	P90	n	P10	P25	P50	P75	P90
Leg Vertical Stiffness (kN/m)	7	13	10.97	11.44	12.90	16.25	16.89	12	8.49	9.52	10.00	11.14	11.91
	9	12	12.14	12.54	13.26	14.86	16.78	12	10.07	10.22	11.10	13.20	13.90
	11	13	13.73	14.00	14.82	16.40	18.78	12	10.82	11.46	12.07	14.69	15.37
	13	13	14.12	15.11	15.71	18.19	19.66	11	11.60	12.88	14.14	15.58	16.43
Vertical Oscillation (m)	7	13	0.03	0.06	0.06	0.07	0.08	12	0.05	0.06	0.07	0.07	0.08
	9	12	0.05	0.06	0.06	0.07	0.08	12	0.06	0.06	0.07	0.07	0.08
	11	13	0.05	0.05	0.06	0.07	0.08	12	0.05	0.06	0.06	0.07	0.07
	13	13	0.05	0.05	0.06	0.06	0.07	11	0.04	0.04	0.06	0.06	0.06
Cadence (steps/min)	7	13	155.52	159.00	159.60	165.00	167.76	12	153.66	155.55	166.50	171.90	176.22
	9	12	155.46	157.80	162.00	165.75	168.36	12	159.78	161.85	171.90	177.45	185.10
	11	13	161.40	163.80	167.40	171.60	174.84	12	168.12	171.00	177.90	181.20	191.10
	13	13	164.52	165.60	173.40	177.60	181.68	11	176.40	178.50	183.60	187.80	207.60

Sensitivity sample													
Variable	Velocity	Males						Females					
		n	P10	P25	P50	P75	P90	n	P10	P25	P50	P75	P90
Leg Vertical Stiffness (kN/m)	7	12	10.93	11.40	12.77	15.16	16.97	11	8.40	9.48	9.91	10.97	11.98
	9	12	12.14	12.54	13.26	14.86	16.78	11	10.05	10.22	11.08	12.61	13.98
	11	12	13.72	13.96	14.61	15.87	17.74	11	10.82	11.26	11.89	14.16	15.38
	13	12	14.00	15.10	15.66	17.43	18.95	11	11.60	12.88	14.14	15.58	16.43
Vertical Oscillation (m)	7	12	0.03	0.06	0.06	0.07	0.08	11	0.05	0.06	0.07	0.08	0.08
	9	12	0.05	0.06	0.06	0.07	0.08	11	0.06	0.06	0.07	0.07	0.08
	11	12	0.05	0.06	0.06	0.07	0.08	11	0.05	0.06	0.06	0.07	0.07
	13	12	0.05	0.05	0.06	0.06	0.07	11	0.04	0.04	0.06	0.06	0.06
Cadence (steps/min)	7	12	155.46	158.25	159.60	165.00	166.62	11	153.60	155.10	168.00	172.80	176.40
	9	12	155.46	157.80	162.00	165.75	168.36	11	159.60	161.70	171.00	179.10	185.40
	11	12	161.10	163.80	167.40	171.15	173.22	11	168.00	170.40	176.40	181.50	192.00
	13	12	164.46	165.45	173.40	176.70	178.68	11	176.40	178.50	183.60	187.80	207.60

Table S2. Percentile estimates of running biomechanical variables for the full and sensitivity samples. Values are presented as P10, P25, P50, P75, and P90. *n* indicates the number of participants at each running speed and sex. The sensitivity sample excludes the two participants with an incomplete four-speed series.
